# PHY906(KD018), an adjuvant based on a 1800-year-old Chinese medicine, enhanced the anti-tumor activity of Sorafenib by changing the tumor microenvironment

**DOI:** 10.1038/srep09384

**Published:** 2015-03-30

**Authors:** Wing Lam, Zaoli Jiang, Fulan Guan, Xiu Huang, Rong Hu, Jing Wang, Scott Bussom, Shwu-Huey Liu, Hongyu Zhao, Yun Yen, Yung-Chi Cheng

**Affiliations:** 1 Department of Pharmacology, Yale University School of Medicine, New Haven, Connecticut 06510, USA; 2 Department of Computational Biology and Bioinformatics, Yale University School of Medicine, New Haven, Connecticut 06510, USA; 3 PhytoCeutica Inc. Branford, Connecticut 06405, USA; 4 Department of Molecular Pharmacology. City of Hope, Duarte, California, USA

## Abstract

PHY906 (KD018) is a four-herb Chinese Medicine Formula. It has been shown to potentially enhance the therapeutic indices of different class anticancer agents *in vivo*. Here, PHY906 is reported to enhance the anti-tumor activity of Sorafenib in nude mice bearing HepG2 xenografts. Among the four herbal ingredients of PHY906, *Scutellaria baicalensis* Georgi (S) and *Paeonia lactiflora* Pall (P) are required; however, S plays a more important role than P in increasing tumor apoptosis induced by Sorafenib with an increase of mouse(m)FasL and human(h)FasR expression. PHY906 may potentiate Sorafenib action by increasing hMCP1 expression and enhancing infiltration of macrophages into tumors with a higher M1/M2 (tumor rejection) signature expression pattern, as well as affect autophagy by increasing AMPKα-P and ULK1-S555-P of tumors. Depletion of macrophage could counteract PHY906 to potentiate the anti-tumor activity of Sorafenib. It was reported that tumor cells with higher levels of ERK1/2-P are more susceptible to Sorafenib, and the S component of PHY906 may increase ERK1/2-P via inhibition of ERK1/2 phosphatase in HepG2 tumors. PHY906 may potentiate the anti-hepatoma activity of Sorafenib by multiple mechanisms targeting on the inflammatory state of microenvironment of tumor tissue through two major ingredients (P and S) of PHY906.

The most common type of liver cancer, hepatocellular carcinoma (HCC) has a 5-year survival rate of only ~14%. HCC patients usually present in advanced stages, at which point surgical resection and/or chemical embolism are no longer feasible^1^. The median prognosis of patients with unresectable and recurrent HCC ranges from 3 to 7 months^2^. Sorafenib, an inhibitor of the RAF/MEK/ERK pathways as well as tyrosine kinase receptors such as VEGF, PDGF, and Kit, is the only FDA-approved drug for the treatment of HCC^3^. The most common side effects of Sorafenib are diarrhea, hand-foot skin reactions, nausea, fatigue, and pain. Unfortunately, the treatment only increases median survival time by 3–4 months^4^; hence, more effective treatments for advanced HCC are needed.

PHY906(KD018) is currently being developed as an adjuvant for chemotherapy. PHY906 (KD018) is based on the Huang Qin Tang herbal mixture, which was first described in Chinese texts 1800 years ago for treatment of numerous gastrointestinal symptoms, including diarrhea, nausea, and vomiting. The mixture consists of four herbs: *Glycyrrhiza uralensis* Fisch (**G**), *Paeonia lactiflora* Pall (**P**), *Scutellaria baicalensis* Georgi (**S**), and *Ziziphus jujuba* Mill (**Z**). PHY906(KD018) was prepared using high-quality herbs picked by experienced herbalists and manufactured according to cGMP (current Good Manufacturing Practice). Consistent preparations of PHY906 have been made over a period of 10 years as demonstrated by Phytomics QC using standardized chemical and biological fingerprints^5^.

In preclinical studies, PHY906 was demonstrated to reduce gastrointestinal toxicity caused by irinotecan (CPT-11) treatment, while enhancing the anti-tumor activity of CPT-11^6^. All four herbs of PHY906 were required to maximally enhance the therapeutic activity of CPT-11 *in vivo*^6^. PHY906 was shown to reduce CPT-11-induced intestinal inflammation by inhibiting the NFκB, COX2, and iNOS pathways^6^. It was also found to promote intestinal progenitor cell repopulation in the damaged intestine by both increasing expression of several components of the Wnt signaling pathway and potentiating wnt3a action^6^. Recently, PHY906 was demonstrated to decrease toxicity from fractionated abdominal irradiation by promoting faster recovery of the intestine^7^. In tumors, PHY906 was shown to increase the anti-tumor activity of CPT11, resulting in an increase in apoptosis and macrophage infiltration. mRNA array study suggests that PHY906 plus CPT-11 alters the gene expression profiles of tumor to become highly inflamed and pro-apoptotic, favoring tumor rejection^8^.

Currently, three phase I/II and one phase II clinical trials for PHY906 have been finished in U.S. In these clinical studies, PHY906 was demonstrated to reduce chemotherapy-associated side effects for patients with metastatic colorectal cancer (mCRC) (PHY906+CPT-11/5FU/LV)^9^,^10^ and advanced pancreatic cancers (PHY906+capecitabine)^11^,^12^. In a phase I/II clinical trial for advanced hepatocellular carcinoma (HCC), PHY906 appeared to decrease capecitabine-associated toxicity^13^. The median patient survival time was 10.9 months, which is comparable to that of patients treated only with Sorafenib in U.S./European trials and much longer than the 7-month median survival seen in Asian Sorafenib trials^13^. Since Sorafenib is the only approved drug for the treatment of HCC, and since diarrhea is a serious side effect of Sorafenib treatment, we used HepG2-bearing nude mice as a model to study the combination effect of PHY906 and Sorafenib on tumor growth. Our results support the possibility that PHY906 might improve the therapeutic index of Sorafenib in patients with advanced liver cancer, a combination currently entering phase I clinical trials in the United States (National Clinical Trial Identifier: NCT01666756).

## Results

### PHY906 enhances the antitumor activity of Sorafenib against HepG2 tumors without affecting the body weight of animals

**Figure 1:**
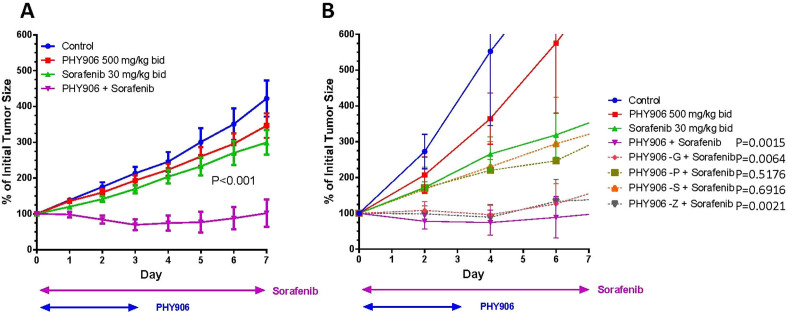
Antitumor activity of PHY906, one herb deleted PHY906, and/or Sorafenib on HepG2 tumor growth in NCR nude mice. (A) Effect of PHY906 and/or Sorafenib on HepG2 (human) tumor growth in NCR nude mice. Error bars indicate standard deviations and N = 14. (B) Effect of one herb deleted of PHY906 and Sorafenib on HepG2 tumor growth in NCR nude mice. Error bars indicate standard deviations and N = 5. Sorafenib (30 mg/kg, b.i.d., daily) and PHY906 (500 mg/kg, b.i.d.) was administered orally from day 0 to day 3. Details of experimental procedures are given in Materials and Methods. T-test was used to compare the difference of Sorafenib vs. PHY906 or one herb deleted of PHY906 at day 7. P values are indicated in the graphs.

For one cycle (7 day) of drug treatment, PHY906 (500 mg/kg, p.o) was fed twice per day from day 0 to day 3 either with or without Sorafenib (30 mg/kg, b.i.d, p.o, daily) to animals harboring HepG2 tumors, and HepG2 tumor growth and nude mice weight loss were studied. Animal body weights were not affected by any treatments (Fig S1A S2B), and HepG2 tumor growth was not affected by PHY906 treatment (P > 0.05) (Fig. 1A). Whereas Sorafenib reduced HepG2 tumor growth by 35% at day 8 as compared to the control group (Fig. 1A), Sorafenib with PHY906 (So+PHY906) completely inhibited HepG2 tumor growth (P < 0.001) and even enhanced the shrinkage of HepG2 tumors during treatment (Fig. 1A). Increasing Sorafenib treatment to 60 mg/kg or 120 mg/kg b.i.d did not enhance its antitumor activity, with or without PHY906 co-treatment (Fig S2A). So+PHY906 could continue inhibit HepG2 tumor growth within two-cycle treatment or three cycle treatment. (Fig S2A S2C). In our study, Sorafenib did not cause histological changes in the small intestine (Fig S3). In the following preclinical studies, we used treatment concentrations of 500 mg/kg (b.i.d) for PHY906 and 30 mg/kg (b.i.d) for Sorafenib.

Comparing the antitumor activity of Sorafenib to that of So+PHY906 minus one herb component (PHY906-**G**, PHY906-**P**, PHY906-**S**, PHY906-**Z**) revealed that deletion of either **P** or **S** eliminated PHY906's synergistic activity with Sorafenib (Fig. 1B). Thus, **P** and **S** are essential to PHY906's enhancement of Sorafenib's antitumor activity.

PHY906 also enhanced the antitumor activity of Sorafenib against Hepa1–6 tumors in both nude and BDF1 mice (immunocompetent mouse) (Fig S1B–D). Therefore, T-cell deficiency is not required for PHY906 to enhance the antitumor activity of Sorafenib.

### PHY906 increases apoptosis in HepG2 tumors treated with Sorafenib

**Figure 2:**
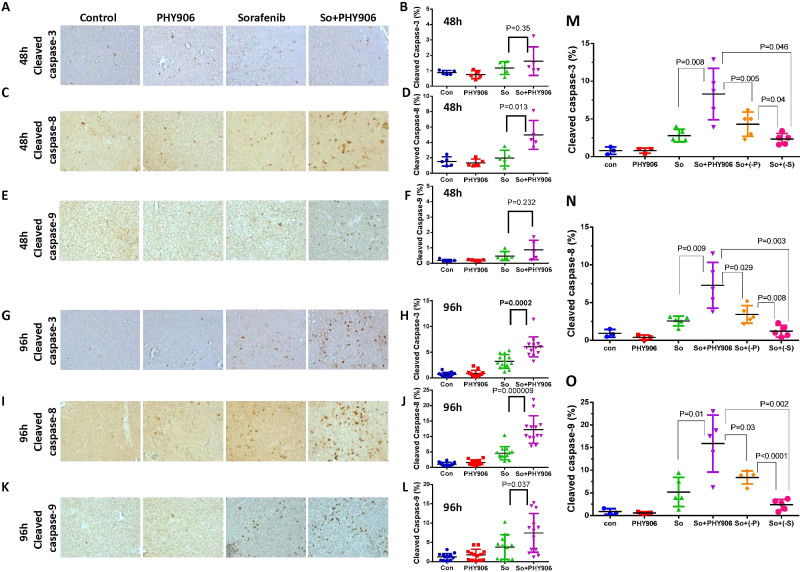
Effect of PHY906, Sorafenib (So), Sorafenib+PHY906 (So+PHY906), Sorafenib+PHY906 deleted P (So+(-P)) and Sorafenib+PHY906 deleted S (So+(-S)) on the induction of apoptosis and growth of HepG2 tumors in NCR nude mice. Immunohistochemistry staining for cleaved caspase-3 (A, G), cleaved caspase-8(C, I), cleaved caspase-9 (E, K) of HepG2 tumor section after treatment with Sorafenib and So+PHY906 for 48 h and 96 h. Percentage of cleaved caspase-3(B, H, M), cleaved caspase-8(D, J, N), cleaved caspase-9 (F, L, O) stained cell per each view of HepG2 tumor section after the drug treatment for 48 h and 96 h. Each spot represents a mean of the number of heavily stained cells from 4 to 5 views of each tumor section against total live cells (PCNA-stained) in each treatment group. Number of animals are 5 to 9. Sorafenib (30 mg/kg, b.i.d.) and/or PHY906, (-P), (-S) (500 mg/kg, b.i.d.) were administered orally twice a day. Details of experimental procedures are given in Materials and Methods.

PHY906 treatment increases apoptosis in Sorafenib-treated HepG2 tumors as indicated by an increase in cleaved caspase-3 at 96 h (P = 0.0002) but not at 48 h (Fig. 2A, 2B, 2G, 2H). Two upstream proteins of caspase-3, cleaved caspase-8 and cleaved caspase-9, were also examined post-treatment. At 48 h, So+PHY906 treatment significantly increased cleaved caspase-8 (P = 0.013) but not cleaved caspase-9 relative to Sorafenib treatment alone (Fig. 2C–F). At 96 h as well, So+PHY906 treatment resulted in more cleaved caspase-8 (P < 0.0001) (Fig. 2I 2J) and cleaved caspase-9 (P = 0.037) (Fig. 2K 2L) than Sorafenib treatment alone. This suggested that both death receptor and mitochondria-mediated apoptosis were enhanced in the combination therapy within 96 hrs. In contrast, one of the DNA damage markers, H2AX-ser139P, was not increased under any treatment conditions by 48 h or 96 h (Fig S4).

Deletion of **P**(P = 0.002) or **S**(P = 0.006) from PHY906 diminished Sorafenib's enhancement of antitumor activity at 96 h post-treatment (Fig S5). Removal of either component was accompanied by significant decreases in cleaved caspase-3 (Fig. 2M, S6), cleaved caspase-8 (Fig. 2N, S6), and cleaved caspase-9 (Fig. 2O, S6) compared to So+PHY906 treatment (All P values < 0.05). Meanwhile, the number of the cleaved caspases of So+(-**S**) were lower than those of So+(-**P**)(All P values < 0.05). These results are consistent with the hypothesis that **S** plays a more important role than **P** in increasing apoptosis of HepG2 tumors when combined with Sorafenib.

### So+PHY906 increases expression of FasL and FasR, potentially triggering apoptosis directly via caspase-8 or indirectly via caspase-9

**Figure 3:**
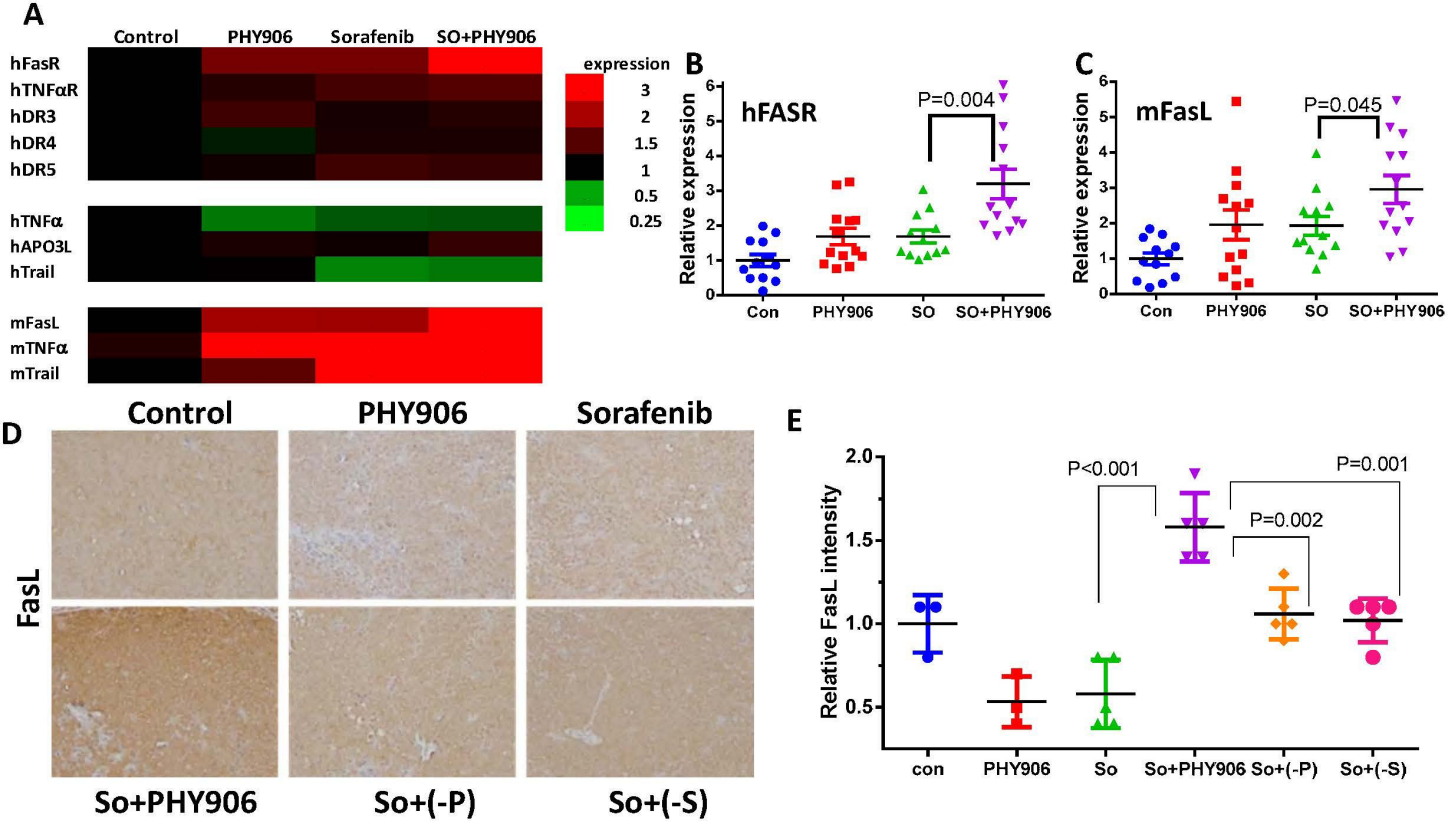
Effect of PHY906 and/or Sorafenib (So) on the expression of death receptors and their ligands level HepG2 tumor in NCR nude mice. (A) Heat map for the mRNA expression levels of death receptors and their ligands in HepG2 tumors after 96 h drug treatment (Each bar represents a mean of two to three different experiments (triplicate samples of each; number of animals for 96 h treatment group is 14). The mRNA expression of human FasR (B) and mouse FasL (C) of HepG2 tumor after the drug treatment for 96 h (Each spot represents a mean of two to three different qRT-PCR experiments (triplicate samples of each; number of animals for 48 h treatment group is 5 and number of animals for 96 h treatment group is 14). (D) Immunohistochemistry staining for FasL protein of HepG2 tumor section after the drug treatments (PHY906, Sorafenib (So), Sorafenib+PHY906 (So+PHY906), Sorafenib+PHY906 deleted P (So+(-P)) and Sorafenib+PHY906 deleted S (So+(-S))) for 96 h. (E) Quantification of immunohistochemistry staining of FasL using imaging software. (Each spot represents a mean of the intensity of FasL staining from 5 views of a tumor section; number of animals for 96 h treatment group is 5). Sorafenib (30 mg/kg b.i.d.) and/or PHY906, (-P), (-S) (500 mg/kg b.i.d.) was administered orally twice daily. Details of experimental procedures are given in Materials and Methods.

Since So+PHY906 treatment increased cleavage of caspase-8, a downstream component of the death receptor pathways, we examined the expression of several death receptors (hFasR, hTNFαR1, hDR3, hDR4, hDR5) and their potential ligands (FasL, TNFα, Apo3L, Trail) using qRT-PCR. Both Sorafenib and So+PHY906 increased hFasR, hTNFαR1, and hDR5 expression in HepG2 cells at 96 h (Fig. 3A). Cells treated with So+PHY906 exhibited significantly higher hFasR than cells treated with Sorafenib alone (P = 0.004) (Fig. 3B). In addition, both Sorafenib and So+PHY906 decreased hTNFα and hTrail, but increased mFasL, mTNFα, and mTrail mRNA expression at 96 h (Fig. 3A). Among those changes, So+PHY906 induced significantly higher expression of mFasL than did Sorafenib (P = 0.045) (Fig. 3C). Immunohistochemistry revealed that So+PHY906 enhanced expression of FasL protein in HepG2 tumor sections as compared to Sorafenib treatment alone (P = 0.00034) (Fig S7A, S7B). Both **P** and **S** were necessary for this increase in FasL: deletion of either **P**(P = 0.002) or **S**(P = 0.001) from PHY906 caused a significant drop in FasL expression relative to So+PHY906 treatment (Fig. 3D, 3E).

### So+PHY906 increases both macrophage infiltration and the M1/M2 (tumor rejection) signature expression pattern in tumor cells

**Figure 4:**
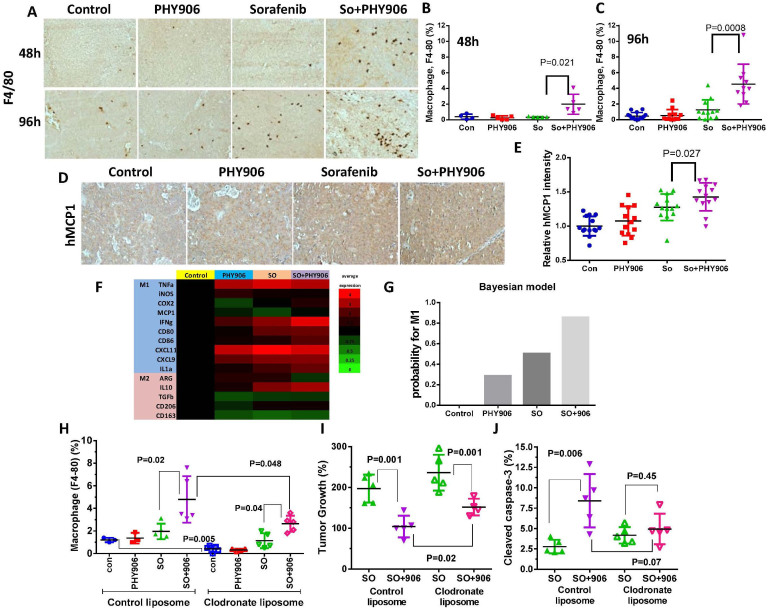
Effect of PHY906 and/or Sorafenib (So) on the infiltration of macrophages in HepG2 tumors in NCR nude mice. (A) Immunohistochemistry staining for F4/80 of HepG2 tumor section after treatment with Sorafenib (So) or So+PHY906 for 48 h and 96 h. Percentage of F4/80 stained cells per view of HepG2 tumor section after the treatments for 48 h (B) and 96 h(C). Each spot represents a mean of the number of F4/80 stained cells from 4 to 5 views of each tumor section against total live cells (PCNA-stained) in each treatment group. (D) Immunohistochemistry of hMCP1 of HepG2 tumor section tumor after PHY906 and/or Sorafenib (So) treatment for 96 h. (E) Quantification of immunohistochemistry staining of hMCP1 using imaging software. (Each spot represents a mean of the intensity of hMCP1 staining from 5 views of a tumor section; N = 14). (F) Heat map for mRNA expression of M1 and M2 macrophage markers in HepG2 tumor at 96 h following the drug treatment (Each bar represents a mean of two to three different qRT- PCR experiments (triplicate samples of each; N = 14). (G) Bayesian analysis for the probability of M1 phenotype. (H) Effect of clodronate liposomes treatment on macrophage infiltration with Sorafenib (So) or So+PHY906 for 96 h. Liposomes were given using i.p. injection at day -2), day 0 and day 2. (I) Effect of clodronate liposomes treatment on HepG2 tumor growth following treatment with Sorafenib (So) or So+PHY906 for 96 h. (J) Effect of clodronate liposomes treatment on apoptosis of HepG2 tumor following treatment with Sorafenib (So) or So+PHY906 for 96 h, N = 5. Sorafenib (30 mg/kg, b.i.d.) and PHY906 (500 mg/kg b.i.d.) were administered orally twice a day. Details of experimental procedures are given in Materials and Methods.

Since macrophage cells could secrete mFasL, the macrophage infiltration of tumor grafts was examined in mice treated with either So+PHY906 or Sorafenib alone. The number of macrophages in HepG2 tumors was increased by So+PHY906 but not Sorafenib as early as 48 h post-treatment (P = 0.021) (Fig. 4A 4B). At 96 h, Sorafenib induced macrophage infiltration but to a lesser extent than So+PHY906(P = 0.0008) (Fig. 4A 4C). So+PHY906 treatment could also induced macrophage infiltration in Hepa 1–6 tumors (murine tumor) in both nude mice and BDF1 mice after 9-day treatment (Fig S8A–C). In contrast to So+PHY906, Sorafenib did not induce macrophage infiltration of Hepa 1–6 tumors in nude mice but reduced macrophage number of Hepa 1–6 tumor in BDF mice (Fig S8A–C). These results suggest that So+PHY906 induced macrophage infiltration of tumors is not restricted to T cell deficit nude mice. However, Sorafenib might reduce macrophage number of tumors when T cells are present.

In order to determine what triggered the macrophage infiltration of HepG2 tumors, we examined mRNA expression of the macrophage-tactic factors MCP1(CCL2), MCSF, MIP1a(CCL3), MIP1b(CCL4), CXCL14, PDGF_BB, Rantes (CCL5), and TNFα. The results indicated that So+PHY906 induced hMCP1 to a significantly higher degree than Sorafenib alone (P = 0.05) (Fig S9A). Immunohistochemistry further validated this finding (P = 0.027) (Fig. 4D 4E). mMIF1a, which triggers macrophage activation, and mIL1a, a product of macrophage activation, were also increased in the So+PHY906 group as compared to Sorafenib-only group (P = 0.006 and P = 0.023, respectively) (Fig S9B S9C). Induction of macrophage infiltration and hMCP1 by So+PHY906 was not observed at 96 h when **P** or **S** were deleted from PHY906 (P values < 0.05 and P < 0.001 respectively) (Fig S10A, S10B, S10C).

Depending on tissue microenvironment and activation pathways, macrophages can be differentiated into two distinct phenotypes: M1 (tumor rejection) and M2 (tumor promotion)^14^. The mRNA expression pattern of M1-associated genes (mTNFα, miNOS, mCOX2, mMCP1, mINFγ, mCD80, mCD86, mCXCL11, mCXCL9, mIL1a) and M2-associated genes (mARG, mIL10, mTGFβ, mCD206, mCD163) (Fig. 4F) were studied in HepG2 tumors and analyzed using a Bayesian model (Fig. 4G) (please see supplementary methods). This analysis suggested that macrophages in tumors treated with So+PHY906 had a nearly 90% chance of possessing the M1 phenotype, a much higher outcome than those in tumors treated with Sorafenib alone (Fig. 4G).

In order to determine if infiltration of macrophage is required for PHY906 to enhance the antitumor activity of Sorafenib, clodronate liposomes were used to deplete macrophages during the treatments. Results indicated that clodronate liposomes could significantly deplete macrophages in HepG2 tumors in comparing to control liposome (P = 0.005) (Fig. 4H, S11A). Under clodronate liposome conditions, PHY906 still enhanced the macrophage infiltration in HepG2 tumors following So+PHY906 treatment (P = 0.04) but the number of macrophage was significantly lower than that of control liposome group (P = 0.048) (Fig. 4H, S11A). Although PHY906 still enhanced the anti-tumor activity of sorafenib under macrophage depleted conditions (P = 0.001) (Fig. 4I), tumors of clodronate liposome/So+PHY906 group are significantly larger than those of control liposome/So+PHY906 group (P = 0.02) (Fig. 4I). In addition, under macrophage depleted conditions, PHY906 no longer significantly enhanced cleaved caspase-3, cleaved caspase-8 and cleaved caspase-9 of HepG2 tumors when co-treated with Sorafenib (P = 0.45, P = 0.13, P = 0.55, respectively) (Fig. 4J, S11B, S11C). Therefore, macrophage infiltration induced by PHY906 plays a key role in enhancing the antitumor activity of Sorafenib against HepG2 tumor growth.

### So+PHY906 increases autophagy associated with AMP-activated protein kinase α (AMPKα) and ULK1 phosphorylation

**Figure 5:**
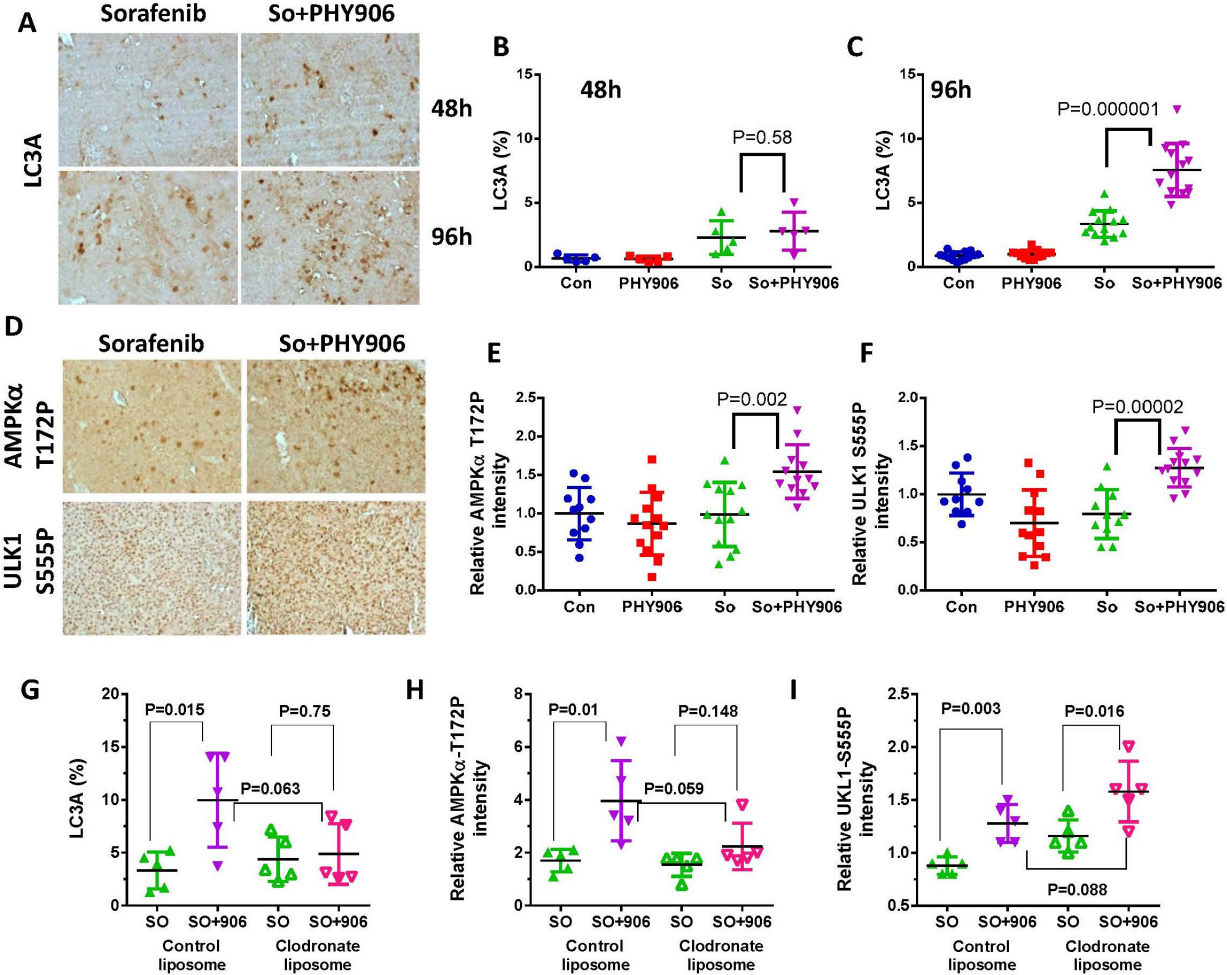
Effect of PHY906 and/or Sorafenib (So) on the autophagy of HepG2 tumors in NCR nude mice. (A) Immunohistochemistry staining for LC3A of HepG2 tumor section after the treatment of Sorafenib and So+PHY906 for 48 h and 96 h. Percentage of LC3A stained cell per each view of HepG2 tumor section after the treatment of PHY906 and/or Sorafenib (So) for 48 h (B) and 96 h(C). Each spot represents a mean of the number of LC3A stained cells from 4 to 5 views of each tumor section against total live cells (PCNA-stained) in each treatment group. The number of animals in the 48 h treatment group is 5 and the number of animals for the 96 h treatment group is 14. (D) Immunohistochemistry staining for AMPKα T172P and ULK1 S555P of HepG2 tumor sections after drug treatments for 96 h. Quantification of immunohistochemistry staining of AMPKα T172P (E) and ULK1 S555P (F) using imaging software. (Each spot represents a mean of the intensity of brown color from 5 views of a tumor section; number of animals for 96 h treatment group is 14). Quantitation of immunohistochemistry staining for LC3A (G), AMPKα T172P (H) and ULK1 S555P (I) of HepG2 tumor sections following treatment of Sorafenib (So) or So+PHY906 with control liposome or clodronate liposome for 96 h. Liposomes were given using i.p. injection at day -2, day 0 and day 2. Each spot represents a mean of the number LC3A stained cells or the intensity of AMPKα T172P and ULK1 S555P staining from 4 to 5 views of each tumor section in each treatment group. Details of experimental procedures are given in Materials and Methods.

In addition to the increase in apoptosis, So+PHY906 also increased autophagy markers in HepG2 tumors at 96 h as indicated by LC3A staining (Fig. 5A–C). There are several pathways leading to autophagy; So+PHY906 treatment had no effect on phosphorylation of either AKT or S6K, which are respectively upstream and downstream of mTOR (Fig S12). ULK S758, which was reported to be dephosphorylated in the case of mTOR inhibition^15^, was not affected by any treatments (Fig S12). Meanwhile, AMPKα-T172-P and ULK1-S555-P, which were reported to be phosphorylated during autophagy^16^, were more highly induced in the So+PHY906 group than in the Sorafenib-only group(P = 0.002 and P = 0.00002, respectively) (Fig. 5D, 5E, 5F). The increase of LC3A and AMPKα-T172-P but not ULK1-S555-P by So+PHY906 could be counteracted by clodronate liposome treatment (Fig. 5G, 5H, 5I). This implies that macrophage infiltration could be an importance factor in promoting autophagy by increasing AMPKα-T172-P of HepG2 tumors under So+PHY906 treatment conditions.

**P** and **S** were found to be equally important in preserving So+PHY906's induction of autophagy marker LC3A since tumor sections from So+PHY906 minus either **P** or **S** exhibited significantly lower levels of LC3A than those treated with complete So+PHY906 at 96 h post-treatment (P values < 0.05) (Fig S13A, S13B). Deletion of **S** or **P** reduced AMPKα-T172-P (P = 0.03 or P = 0.06, respectively) as compared to AMPKα-T172-P of So+PHY906 (Fig S13A, S13C). **P** and **S** were also both required for So+PHY906 to increase expression of ULK1-S55P (Fig S13A, S13D).

### PHY906 increases ERK1/2 phosphorylation by inhibiting ERK1/2 phosphatase

**Figure 6:**
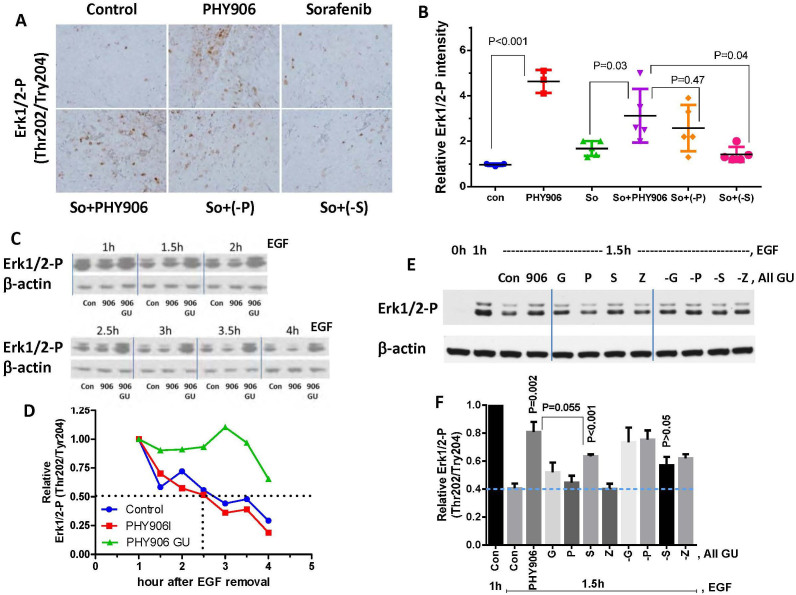
Effect of PHY906, Sorafenib (So), Sorafenib+PHY906 (So+PHY906), Sorafenib+PHY906 deleted P (So+(-P)), and Sorafenib+PHY906 deleted S (So+(-S)) on Erk1/2 phosphorylation of HepG2 tumors in NCR nude mice. (A) Immunohistochemistry staining for phosphorylated Erk1/2 (Thr202/Tyr204) in HepG2 tumor sections after the drug treatment for 96 h. (B) Quantification of immunohistochemistry staining of Erk1/2 phosphorylation using imaging software. (Each spot represents a mean of the intensity of brown color from 5 views of a tumor section; number of animals is 5). (C) Western blotting analysis for the effect of PHY906 or *E.coli* β-glucuronidase treated PHY906 (500 μg/ml) on dephosphorylation rate of Erk1/2 in HepG2 cells following stimulation with EGF (50 ng/ml). β-actin was used as the loading control for normalization. Cropped blots are used in this figure and they have been run under the same experimental conditions (please see the full-length bolts in Fig S16A) (D) Quantification of the Western blot results for the phosphorylated Erk1/2 (Thr202/Tyr204). (E) Western blotting analysis for the effect of *E.coli* β-glucuronidase treated PHY906 (500 μg/ml), equivalent concentration of single herbs (G, P, S, Z), or equivalent concentration of a one herb deleted formula (-G, -P, -S, -Z) on dephosphorylation rate of Erk1/2 in HepG2 cells following stimulation with EGF (50 ng/ml). Cropped blots are used in this figure and they have been run under the same experimental conditions (please see the full-lenght bolts in Fig S16B) (F) Quantification of the Western blot results for the phosphorylated Erk1/2 (Thr202/Tyr204). Details of experimental procedures are given in Materials and Methods.

Previous studies by others indicated that HCC tumors in patients or HCC cells in culture with higher levels of ERK1/2 phosphorylation are more susceptible to Sorafenib^17^,^18^. Here, we found that PHY906 increased ERK1/2 phosphorylation in HepG2 tumors at 48 and 96 h post-treatment (Fig S14). ERK1/2 phosphorylation induced by PHY906 was not completely blocked by Sorafenib at 48 and 96 h (Fig S14). Removing **S** (P = 0.04) but not **P** (P = 0.47) from PHY906 abolished this increase in Erk1/2 phosphorylation (Fig. 6A, 6B). Clodronate liposome treatment did not reduce ERK1/2 phosphorylation under So+PHY906 treatment conditions (Fig S15).

We further examined if PHY906 could have impact on ERK1/2 phosphorylation in cell culture. Since β-glucuronidase treatment could affect PHY906's activity on different signal pathways^6^, we also compared PHY906 with or without β-glucuronidase treatment on ERK1/2 phosphorylation in HepG2 cell. Results indicated that β-glucuronidase-treated PHY906 reduced the dephosphorylation rate of ERK1/2-P but not p38-P, SAPK/JNK-P, or ERK5-P following EGF or H_2_O_2_ treatment (Fig. 6C, 6D, S16A and S18A–C). β-glucuronidase itself did not affect the dephosphorylation rate of ERK1/2-P (Fig S17A S17B).This suggests that β-glucuronidase-treated PHY906 is quite selective in inhibiting ERK1/2 phosphatase(s). β-glucuronidase-treated **S** had a significant inhibitory effect on ERK1/2 phosphatase(s) (P < 0.001: S vs Con at 1.5 h) (Fig. 6E, 6F and S16B) but not as strong as β-glucuronidase-treated PHY906 (P = 0.055: S vs PHY906 at 1.5 h). Deletion of **S** but no other herbs from PHY906 could abolish the ERK1/2 phosphatase(s) inhibitory effect (P > 0.05: **-S** vs Con at 1.5 h) (Fig. 6E, 6F and S16B). This result suggests that **S** plays the most importance role of PHY906 in inhibiting and ERK1/2 phosphatase(s). Other herbs may also contribute some extent to ERK1/2 phosphatase(s) inhibition. Immunohistochemical staining data corroborated these results (Fig. 6A, 6B), demonstrating that deletion of **S** from So+PHY906 did not increase Erk1/2 phosphorylation in HepG2 tumors. The chemicals and ERK1/2 phosphatase(s) involved this process is currently being investigated.

## Discussion

PHY906 enhances the antitumor activity of Sorafenib against hepatoma growth *in vivo*. Combining PHY906 with either Sorafenib or CPT-11 resulted in increased tumor apoptosis^8^. Moreover, the **S** and **P** components of PHY906 are crucial to PHY906's enhancement of both Sorafenib and CPT-11's antitumor activity^19^. **S** was found to be more important than **P** for So+PHY906's induction of apoptosis, though both components are crucial for the apoptosis phenotype. This agree with our previous study that found **S** had stronger activity than **P** in enhancing CPT-11's antitumor activity^19^. Single herb **S** or **P** had much lower activity than full formulation PHY906 in enhancing the action of CPT-11 against tumor growth^19^.

So+PHY906 differentially increased human FasR in tumor cells and murine FasL in host cells. Both **P** and **S** were found to be important for inducing FasL protein expression. As the Fas receptor and ligand were both up-regulated, Fas-mediated cell death pathways could be synergistically activated. This hypothesis is supported by previous studies in which Vorinostat (histone deacetylase inhibitor) was demonstrated to promote sorafenib in killing multiple colon cancer cell lines and HepG2 cells by increasing FasR (CD95) activation and/or expression of FAS ligand in an NFκB-dependent fashion^20^,^21^. Indeed, more cleaved caspase-8 (downstream of death receptors) was detected in tumors following So+PHY906 treatment. It is possible that So+PHY906 indirectly induces cleavage of caspase-9 in HepG2 tumors by increasing caspase-8 expression. Tumor macrophage infiltration was another commonly observed result of both So+PHY906 and CPT-11+PHY906 treatment^8^. This macrophage infiltration phenomenon was also observed when So+PHY906 was administered to nude mice or BDF mice bearing Hepa 1–6 tumors, suggesting that the macrophage infiltration caused by So+PHY906 does not require T-cells. MCP1 is the cytokine responsible for the tumor macrophage infiltration phenotype in mice treated with So+PHY906 and CPT11+PHY906. We further demonstrate that macrophage infiltration plays a key role for PHY906 to enhance the antitumor activity of Sorafenib against HepG2 tumor growth. We also demonstrated that **P** and **S** were essential for PHY906's induction of hMCP1, which, in the presence of Sorafenib, may help attract macrophages to tumors. A biostatistical model suggests that So+PHY906 treatment induces a tumor environment with an increased M1 signature, supporting the presence of M1 macrophages that are believed to have tumor rejection activity^14^. It should be notice that PHY906 could inhibit intestine inflammation triggered by CPT11 by inhibiting many inflammation pathways including NF-κB, COX2 and iNOS^6^ while PHY906 could converse tumor inflammation from chronic status to acute status associated with strong macrophage infiltration following CPT-11^8^ and Sorafenib treatment. Indeed, PHY906 could have different impacts on gene expression on different tissues^8^. According to our clinical studies, only few compounds of PHY906 can be passed thought the intestine and get into plasma in which many new metabolites of PHY906 were detected^22^. This could partly explain why intestine and tumor tissues had different inflammation responses to PHY906.

Autophagy helps recycle unused cellular material to prolong survival under starvation conditions^23^. Recently, autophagy has also been found to promote cell death in extremely harsh conditions and is resultantly being considered as a novel chemotherapeutic target^24^. Sorafenib has been reported to induce autophagy in different HCC cell lines, including HepG2^25^. Here, PHY906 was demonstrated to enhance Sorafenib-induced autophagy, possibly by increasing the phosphorylation of AMPKα-T172-P and ULK1-S555-P. In addition, macrophage infiltration could be involved for autophagy via AMPKα-T172-P. **P** and **S** were found to play critical roles in this process.

It has previously been found that HCC tumors expressing higher levels of ERK1/2 phosphorylation are more susceptible to Sorafenib treatment both *in vitro* and *in vivo*^17^,^18^. Here, we demonstrate that PHY906 treated with recombinant β-glucuronidase increases ERK1/2 phosphorylation in HepG2 tumors by inhibiting ERK1/2 phosphatase(s). Potential targets of PHY906 include DUSP5, DUSP6, DUSP7, and DUSP9, which are reported to selectively dephosphorylate ERK1/2^26^. Prolonged ERK activation is believed to promote the antitumor activity of many chemotherapeutic agents^27^ through Fas/caspase-8-mediated apoptosis^28^,^29^,^30^. The strong Erk1/2 phosphatase inhibitory activity of **S** may help to explain why **S** plays a more important role than **P** in So+PHY906-triggered apoptosis. In addition, ERK activation is reported to be required for LC3-II formation^31^, induction of beclin 1^31^ and BNIP-3^32^, and activation of the RGS19-Gαi3-AGS3 signaling cascade^33^ during autophagy. Hence, prolonged ERK1/2 phosphorylation by PHY906 may facilitate Sorafenib-induced autophagy.

In conclusion, PHY906 is able to potentiate the antitumor activity of Sorafenib through multiple mechanisms. First, So+PHY906 could enhance both apoptosis via up-regulation of the Fas/Fas ligand pathway and tumor autophagy via activation of AMPK pathway. Second, PHY906 could increase Erk1/2 phosphorylation which may facilitate Fas/caspase-8-mediated apoptosis^28^,^29^,^30^ and tumor autophagy^31^,^32^,^33^. Third, PHY906 could also potentiate Sorafenib action by up-regulation of the expression of MCP1 which could attract M1 type macrophages to help rejecting tumors. Both **P** and **S** are essential components of PHY906, but **S** plays a more important role in enhancing Sorafenib's induction of apoptosis and Erk1/2 phosphorylation. We propose that dead tumor cells produce local inflammatory substances that interact with PHY906, stimulating the inflammatory process and ultimately triggering infiltration of macrophages with the M1 type phenotype. Thus, PHY906 may potentiate various antitumor agents that work synergistically in the tumor microenvironment. Based on this preclinical study, a phase I/II clinical trial has been initiated for the treatment of HCC in patients using Sorafenib with a PHY906 adjuvant in US.

## Methods

### Animal Studies

HepG2 cells (1–2 × 10^6^ cells in 0.1 ml phosphate-buffered saline) were transplanted subcutaneously into six-week-old female NCR nude mice (Charles River Laboratories, Wilmington, MA). After 10–14 days, mice with tumor sizes of 150–300 mm^3^ were selected. Tumor volume was estimated by using the formula length × width^2^ × π/6. Unless otherwise indicated, treatment groups each consisted of five mice. Tumor size, body weight, and mortality of the mice were monitored daily. For one cycle of drug treatment, PHY906 was administered orally (p.o.) twice a day for four days (b.i.d., 500 mg/kg) at approximately 10:00am and 3:00pm each day, while Sorafenib was administered orally (p.o.) twice a day for seven days (b.i.d., 30 mg/kg) at approximately 10:00am and 3:00pm each day. On Day 0, PHY906 was administered 30 minutes prior to Sorafenib administration. In the control groups, mice were administered water for oral administration. PHY906 lot number 10 was used for all animal experiments and cell culture experiments. PHY906 lot number 10 has been used in our previous clinical trial^22^ and its chemical profile has been reported^22^. Chemical profile of PHY906 lot number 10 is very similar to other lots of PHY906 as indicated by Phytomics Similarity Index (PSI) > 0.9 (1, perfectly identical; 0, no similarities) (Please see supplementary table 1). Clodronate or control liposomes (5 mg/ml) given using i.p. injection at day -2(0.4 ml/mouse), day 0 and day 2 (0.2 ml/mouse). Immunohistochemistry was used to detect protein expression in tumor tissues. qRT-PCR was used to quantify mRNA expression. HepG2, was obtained from American Type Culture Collection (American Type Culture Collection, Manassas, VA) on 2003 without further authenticated. Animal experimental protocols were approved by Yale University Institutional Animal Care and Use Committee (IACUC). All animal experiments were carried out in accordance with an approved Yale University Institutional Animal Care and Use Committee (IACUC) protocol.

### Immunohistochemistry

Mice (NCR nude bearing HepG2 tumors) were terminated by cervical dislocation at 48 h or at 96 h after initiation of drug treatment (see above). Tumor and intestinal tissues were removed, fixed in formalin, embedded in paraffin, and sectioned into 10μm. The sections were mounted on Superfrost slides, deparaffinized with xylene, and gradually rehydrated. Antigen retrieval was achieved by 10 mM sodium citrate pH6.0 with 0.02% Tween-20 under steam for 30 minutes. The primary antibodies were diluted using Tris-HCl buffer containing 1% BSA and 0.5% Tween-20 and treated slides were incubated at room temperature for one hour. As a negative control, a set of slides was processed without primary antibody. Super-picture immunohistochemistry detection kit (Life Technologies, Inc. Norwalk, CT) was used for detection. The slides were counterstained with hematoxylin and mounted. These antibodies were used: Cleaved Caspase-3(#9664, Cell Signaling Technology, Inc.), Cleaved Caspase-8(#9496, Cell Signaling Technology, Inc. Danvers, MA), Cleaved Caspase-9(#ab52298, Abcam, Cambridge, England), FASL(#sc-834, Santa Cruz Biotechnology, Inc. Dallas, TX), F4/80(#ab16911, Abcam), hMCP1(#ab9669, Abcam), LC3A/B(#4108, Cell Signaling Technology, Inc.), Phospho-AMPKα(Thr172) (#2535, Cell Signaling Technology, Inc.), Anti-phospho-ULK1(Ser555)(#ABC124, Millipore, Billerica, MA), Phospho-p44/42 MAPK(ERK1/2) (#4376, Cell Signaling Technology, Inc.), CD34(#ab8158, Abcam).

### *E.coli* β-glucuronidase treatment

PHY906 or other herbal extracts (50 mg/ml,Tris-HCl 100 mM,pH 6.8) were incubated with recombinant E.coli β-glucuronidase with 6xHIS at C-terminal (40 μg/ml) at 37°C in a water bath for one hour. After incubation, herbal extracts was sterilized by boiling at 100°C for 5 min.

### Western Blotting

HepG2 Cells were lysed in 2× SDS sample buffer (62.5 mM Tris-HCl, 2% SDS, 10% glycerol, 50 mM DTT, and 0.05% bromophenol blue) and sonicated for 10 s to shear DNA. The whole-cell extracts were then electrophoresed through 12% SDS-polyacrylamide gels and transferred to nitrocellulose membranes (Bio-Rad Laboratories, Inc) with a Mini PROTEANII transfer apparatus (Bio-Rad). The membranes were blocked and probed in TBS-T buffer (1× Tris-HCl 50 mM pH7.6, NaCl 150 mM, 0.2% Tween-20) containing 5% non-fat milk. Primary antibodies (Phospho-p44/42 MAPK (Erk1/2) (Thr202/Tyr204) (D13.14.4E) XP® Rabbit mAb #4370, Phospho-SAPK/JNK (Thr183/Tyr185) (81E11) Rabbit mAb #4668, Phospho-p38 MAPK (Thr180/Tyr182) (D3F9) XP® Rabbit mAb #4511, Cell Signaling Technology, Inc.) at 1:1000 in TBS-T were incubated with the membrane overnight at 4°C. β-actin was used as an internal control for normalization and detected with a monoclonal actin antibody diluted at 1:2500 (Sigma, St. Louis, MO). After washing with TBS-T, the membranes were then further incubated with horseradish peroxidase-conjugated anti-mouse IgG and anti-rabbit IgG (1:5000; Sigma), The immunoreactive bands were visualized by enhanced chemiluminescence reagents (Perkin-Elmer Life Science Products, Boston, MA), and densitometry scanning was performed with the densitometer (Molecular Dynamics).

### Quantitative Real-Time PCR

Total RNA was isolated using a modified TRIzol® protocol. Following the manufacturer's instructions, we collected the aqueous phase and then added ethanol. Before centrifuging, this slurry was added to a column (miRNeasy, Qiagen, Venlo, Limburg) for further extraction and simultaneous DNA digestion (RNase-Free DNAse set, Qiagen). cDNA was synthesized using random primers and reverse transcriptase MMLV (New England Biolabs, Ipswich, MA). qPCR assays were performed using iTaq™ SYBR® Green Supermix and the CFX96 Real-Time PCR Detection System (Bio-Rad Laboratories, Hercules, CA). Primer sets were listed in the supplementary method.

### Statistical analysis

Data were analyzed by one-way or two-way ANOVA (GraphPad Prism 4, San Diego, CA), Student's T-test (Microsoft Office Excel). Differences were considered statistically significant when P < 0.05.

## Author Contributions

W.L. did Erk1/2 phosphorylation experiments, took photos for immunohistochemical staining, did qRT-PCR, analyzed results and wrote the manuscript. Z.L. and S.B. did animal experiments. F.L. did immunohistochemical staining. X.H. and H.Z. did statistical analysis for M1, M2 macrophages. J.W. and R.H. did qRT-PCR. S.L. and Y.Y. provided PHY906 and Sorafenib and discussed results. Y.C. designed experiments, analyzed results and wrote the manuscript. All authors reviewed the manuscript.

## Supplementary Material

Supplementary Information PHY906 supplementary methods and figures
