# Supplementary material for: PHY906(KD018), an adjuvant based on a 1800-year-old Chinese medicine, enhanced the anti-tumor activity of Sorafenib by changing the tumor microenvironment
Source: Sci Rep. 2015 Mar 30;5:9384. doi: 10.1038/srep09384 (PMC4377583; doi:10.1038/srep09384)
Supplement: Supplementary Information — PHY906 supplementary methods and figures [file srep09384-s1.pdf]

PHY906(KD018), an adjuvant based on a 1800-year-old Chinese medicine, enhanced the anti-tumor activity of Sorafenib by changing the tumor microenvironment

Wing Lam[1+], Zaoli Jiang[1+], Fulan Guan[1+], Xiu Huang[2], Rong Hu[1], Jing Wang[1], Scott Bussom[1], Shwu-Huey Liu[3], Hongyu Zhao[2], Yun Yen[4] and Yung-Chi Cheng[1]*

[1]Department of Pharmacology
[2]Department of Computational Biology and Bioinformatics
Yale University School of Medicine, New Haven, Connecticut 06510, USA
[3]PhytoCeutica Inc. Branford, Connecticut 06405, USA
[4] Department of Molecular Pharmacology. City of Hope, Duarte, California, USA.

Supplementary Method

Table. 1 Comparison of different lot of PHY906 and HQT-F (commercial *Huang-Qin Tang)* using Phytomics Similarity Index (PSI).

	MS	UV
PHY906-6	1	1
PHY906-7	0.99	0.98
PHY906-8	0.99	0.99
PHY906-10	0.97	0.96
HQT-F	0.81	0.76

MS = PSI determined by comparison of mass spectrum of different lot of PHY906.

UV = PSI determined by comparison of UV spectrum of different lot of PHY906.

where 1=completely the same, 0=completely different

Table. 2 qPCR primers list.

	Forward primer	Reverse primer
hActin	GCCACGGCTGCTTCCAGCTCC	TTGTGCTGGGTGCCAGGGCAGTGA
mActin	TCCTTCTTGGGTATGGAATCCT	TTGTGCTGGGTGCCAGGGCAGTGA
hFasR	AGCTTGGTCTAGAGTGAAAA	GAGGCAGAATCATGAGATAT
hTNFaR	TGCCTACCCCAGATTGAGAA	ATTTCCCACAAACAATGGAGTAG
hDR3	CAAGGCGAAGAAGCACGAAC	GCCGAGAAGTTGAGAAATGTCT
hDR4	CTGATGAAATGGGTCAACAA	AACTTTCCAGAGTCCACCAA
hDR5	AAGACCCTTGTGCTCGTTGT	CCAGGTGGACACAATCCCTC
hTNFa	CCTGCCCCAATCCCTTTATT	CCCTAAGCCCCCAATTCTCT
hAPO3L	TGTTGATTCTGGCTTCCTCC	ACCTAAAGGCCGGAAAACAC
hTrail	GAGCTGAAGCAGATGCAGGAC	TGACGGAGTTGCCACTTGACT
mFasL	GTGGGGGTTCCCTGTTAAAT	ACTCCGTGAGTTCACCAACC
mTrail	TGGAGTCCCAGAAATCCTCA	TCACCAACGAGATGAAGCAG
hMCP1(CCL2)	GTCTCTGCCGCCCTTCTGT	TTGCATCTGGCTGAGCGAG
hMCSF	CCAGCAACTGGAGAGGTGTC	GCAGCTGCAGGAACTCTCTT
hMIF1a	GAAGGCCATGAGCTGGTC	GGTTCCTCTCCGAGCTCAC
hMIP1a(CCL3)	ACAGTGTGTTTGTGATTGTTTGCTC	AGCCACTCGGTTGTCACCAG
hMIP1b(CCL4)	GATTCACTGGGATCAGCACA	CTTTTCTTACACCGCGAGGA
hRantes(CCL5)	CAGGGCTCTGTGACCAGGAA	ACGGCGGAAGCTTAAGAGT
mMCP1(CCL2)	GGCTGGAGCATCCACGTGTT	TGGTGACAAAAACTACAGC
mMCSF	GGTAGTGGTGGATGTTCCCA	CCAGGATGAGGACAGACAGG
mMIF1a	TGACTTTTAGCGGCACGAAC	GACTCAAGCGAAGGTGGAAC
mMIP1a(CCL3)	GTGGAATCTTCCGGCTGTAG	ACCATGACACTCTGCAACCA
mMIP1b(CCL4)	AGAAACAGCAGGAAGTGGGA	GCTCTGTGCAAACCTAACCC
mCXCL14	TTTGGTGCTCTGCAGCTTAG	AGCCAAAGTACCCACACTGC
mRantes(CCL5)	CCACTTCTTCTCTGGGTTGG	GTGCCCACGTCAAGGAGTAT
miNOS	CAGAAGCAGAATGTGACC	GTAGTAGTAGAATGGAGATAGG
mCOX2	TCTCCCCTCTCTACGCATTCTA	ACGGATTGGAAGTTCTATTGGC
mMCP1	CGGAACCAAATGAGATCAGAACCTAC	GCTTCAGATTTACGGGTCAACTTCAC
mIFNg	TGTCTTTCTTGTCTTCAGAAA	TTTCTGAAGACAAGAAAGACA
mCD80	ACCCCCAACATAACTGAGTCT	TTCCAACCAAGAGAAGCGAGG
mCD86	TGTTTCCGTGGAGACGCAAG	TTGAGCCTTTGTAAATGGGCA
mCXCL11	GGCTGCGACAAAGTTGAAGTGA	TCCTGGCACAGAGTTCTTATTGGAG
mCXCL9	GGAGTTCGAGGAACCCTAGTG	GGGATTTGTAGTGGATCGTGC
mIL1a	TGAGTTTTGGTGTTTCTGGC	TCGGGAGGAGACGACTCTAA
mARG	TGAGAGACCACGGGGACCTG	GCACCACACTGACTCTTCCATTC
mIL10	CAGGACTTTAAGGGTTACTTGGGTTG	GCTCCACTGCCTTGCTCTTATTTTC
mTGFb	GTGTGGAGCAACATGTGGAACTCTA	TTGGTTCAGCCACTGCCGTA
mCD206	TCTCCCGGAACCGACTCTTC	GGTCGAGCACATAGGTCTTCT
mCD163	CCTCCTCATTGTCTTCCTCCTGTG	CATCCGCCTTTGAATCCATCTCTTG

Statistical Modeling and Inference of M1/M2 Ratio using EM Algorithm

A normal mixture model is adopted to infer M1/M2 ratio in each sample. We assume that each sample is independent and contains genes from two groups: M1 and M2. For simplicity, we also assume that every gene across all samples shares the same distribution, the expression of which only affected by the M1/M2 state of the sample. Since the M1 and M2 state distribution of each gene is unknown, the problem of estimating the model parameters can be seen as a missing data problem. We therefore use EM algorithm to infer the missing data and model parameters in an iterative manner.

The qPCR detected gene expression levels for each sample i are denoted as $\{g_1, \ldots, g_n\}$, n is the total number of genes selected. We model the gene expression as a mixture of two normal models, denoted by M_k, $k = 1,2$. Let

$$\pi_k = P(g_j \in M_k), j = 1, \ldots, n,$$

where $\pi_k \in (0,1)$ for $k = 1,2$, $\sum_{k=1}^{2} \pi_k = 1$ and g_j represents the expression level for gene j. Conditional on M_k, the gene expression level follows a normal distribution, $g_j \mid_{g_j \in M_k} \sim N(\mu_{jk}, \sigma_{jk})$.

EM algorithm can iteratively update model parameters from the data. The iteration comprises two steps: the Expectation step and the Maximization step. The E-step will calculate the membership value of M_k for each sample i as

$$\hat{p}_{k,i}^{(t)} = \frac{\hat{\pi}_k^{(t-1)} \prod_{j=1}^{n} f\left(g_{ij} \mid \hat{\mu}_{kj}^{(t-1)}, \hat{\sigma}_{kj}^{(t-1)}\right)}{\sum_{l=1}^{2} \hat{\pi}_l^{(t-1)} \prod_{j=1}^{n} f\left(g_{ij} \mid \hat{\mu}_{lj}^{(t-1)}, \hat{\sigma}_{lj}^{(t-1)}\right)},$$

which can be seen as the probability of sample i being in the M_k model. The M-step will update $\hat{\pi}_k^{(t)}$, $\hat{\mu}_{kj}^{(t)}$, and $\hat{\sigma}_{kj}^{(t)}$ respectively based on the expected membership values calculated in the E-step (N is the total number of samples):

$$\hat{\pi}_k^{(t)} = \frac{\sum_{i=1}^{N} \hat{p}_{k,i}^{(t)}}{N}, k = 1,2.$$

$$\hat{\mu}_{kj}^{(t)} = \frac{\sum_{i=1}^{N} \hat{p}_{ki}^{(t)} g_{ij}}{\sum_{i=1}^{N} \hat{p}_{k,i}^{(t)}}, k = 1,2.$$

$$\hat{\sigma}_{kj}^{(t)} = \frac{\sum_{i=1}^{N} \hat{p}_{ki}^{(t)} (g_{ij} - \hat{\mu}_{kj}^{(t)})^2}{\sum_{i=1}^{N} \hat{p}_{k,i}^{(t)}}, k = 1,2.$$

To start the algorithm, initial values are set incorporating known attributes of selected genes' expression tendency under M1 and M2 state:

$$\hat{\mu}_{1,j}^{(0)} = \begin{cases} \frac{\sum_{i=1}^{N} g_{ij}}{N} + 1, \text{if gene } j \text{is M1 marker gene} \\ \frac{\sum_{i=1}^{N} g_{ij}}{N} - 1, \text{if gene } j \text{is M2 marker gene} \end{cases}$$

$$\hat{\mu}_{2,j}^{(0)} = \begin{cases} \frac{\sum_{i=1}^{N} g_{ij}}{N} - 1, \text{if gene } j \text{is M1 marker gene} \\ \frac{\sum_{i=1}^{N} g_{ij}}{N} + 1, \text{if gene } j \text{is M2 marker gene} \end{cases}$$

$$\hat{\sigma}_{kj}^{(0)} = \frac{\sum_{i=1}^{N} (g_{ij} - \mu)^2}{N}, \mu = \frac{\sum_{i=1}^{N} g_{ij}}{N}.$$

$$\hat{\pi}_1^{(0)} = 0.8, \hat{\pi}_2^{(0)} = 0.2.$$

E-step and M-step are repeated until no significant changes occur in the mean square difference of estimated parameters, this is, stop the tth iteration if

$$\frac{\sum_{k} \sum_{j} (\hat{\mu}_{kj}^{(t)} - \hat{\mu}_{kj}^{(t-1)})^2 + \sum_{k} \sum_{j} (\hat{\sigma}_{kj}^{(t)} - \hat{\sigma}_{kj}^{(t-1)})^2 + \sum_{k} (\hat{\pi}_{k}^{(t)} - \hat{\pi}_{k}^{(t-1)})^2}{4n + 2} < \delta,$$

where δ is a sufficiently small number.

For each sample i, the probability of being in state M1 is therefore inferred from the algorithm as $\hat{p}_{1,i}^{(t)}$. The overall probability of samples under certain treatment being in state M1 is calculated as the average of $\hat{p}_{1,i}^{(t)}$ for all the samples under that treatment.

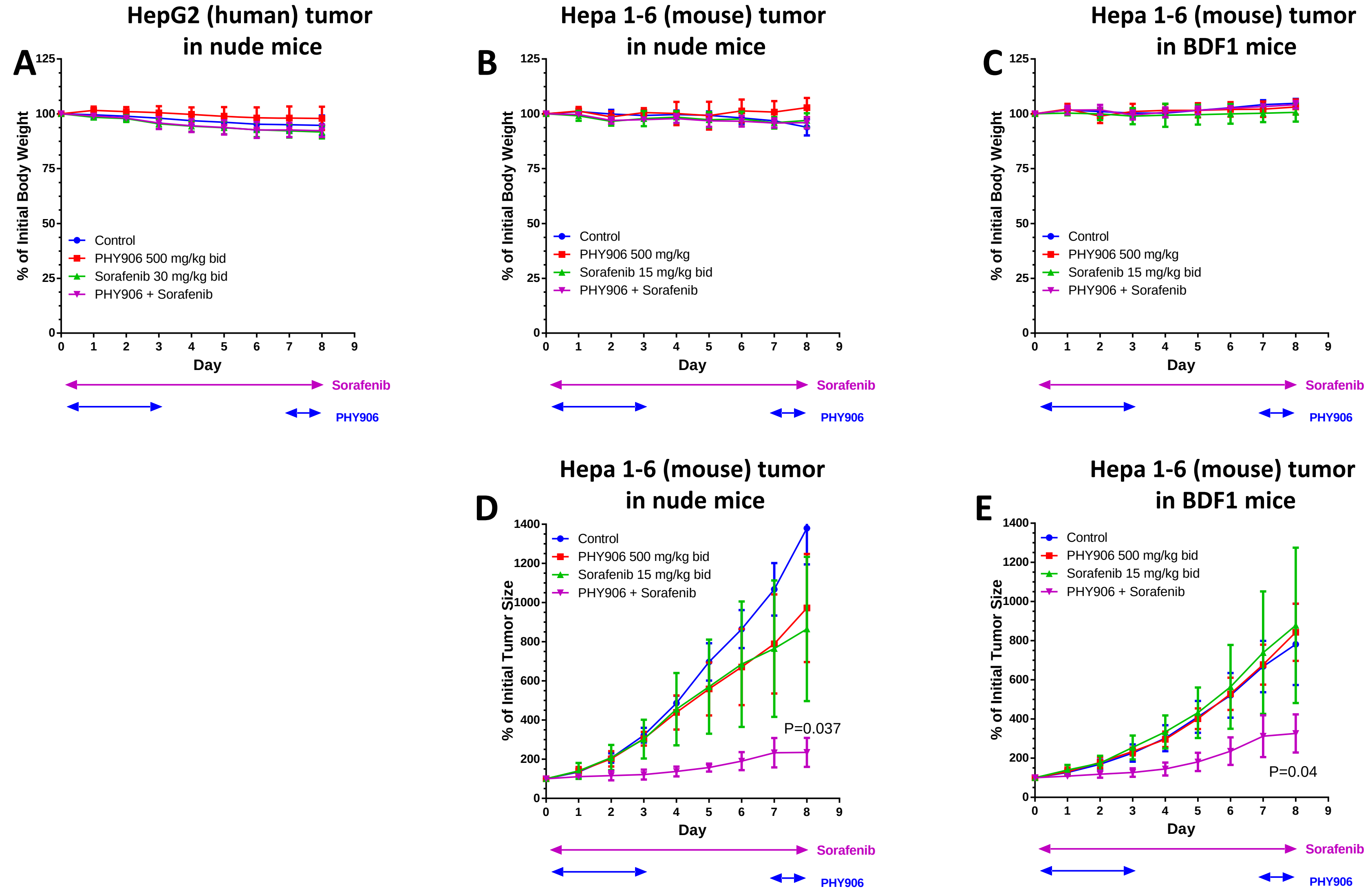

Supplementary Figure 1. Effect of PHY906 and Sorafenib on the body weight of HepG2 tumor bearing NCR nude mice (**A**), Hepa1-6 tumor bearing NCR nude mice (**B)**, Hepa1-6 tumor bearing BDF-1 mice (**C**) and Hepa 1-6 tumor growth in NCR nude mice **(D)** and BDF1 mice (**E**). Sorafenib (30mg/kg or 15mg/kg as indicated in the graphs, b.i.d) was administered orally daily and PHY906 (500mg/kg, b.i.d.) was administered orally from day 0 to day 3 and from day 7 to day 8. Error bars indicate standard deviations and N=14. Details of experimental procedures are given in Materials and Methods

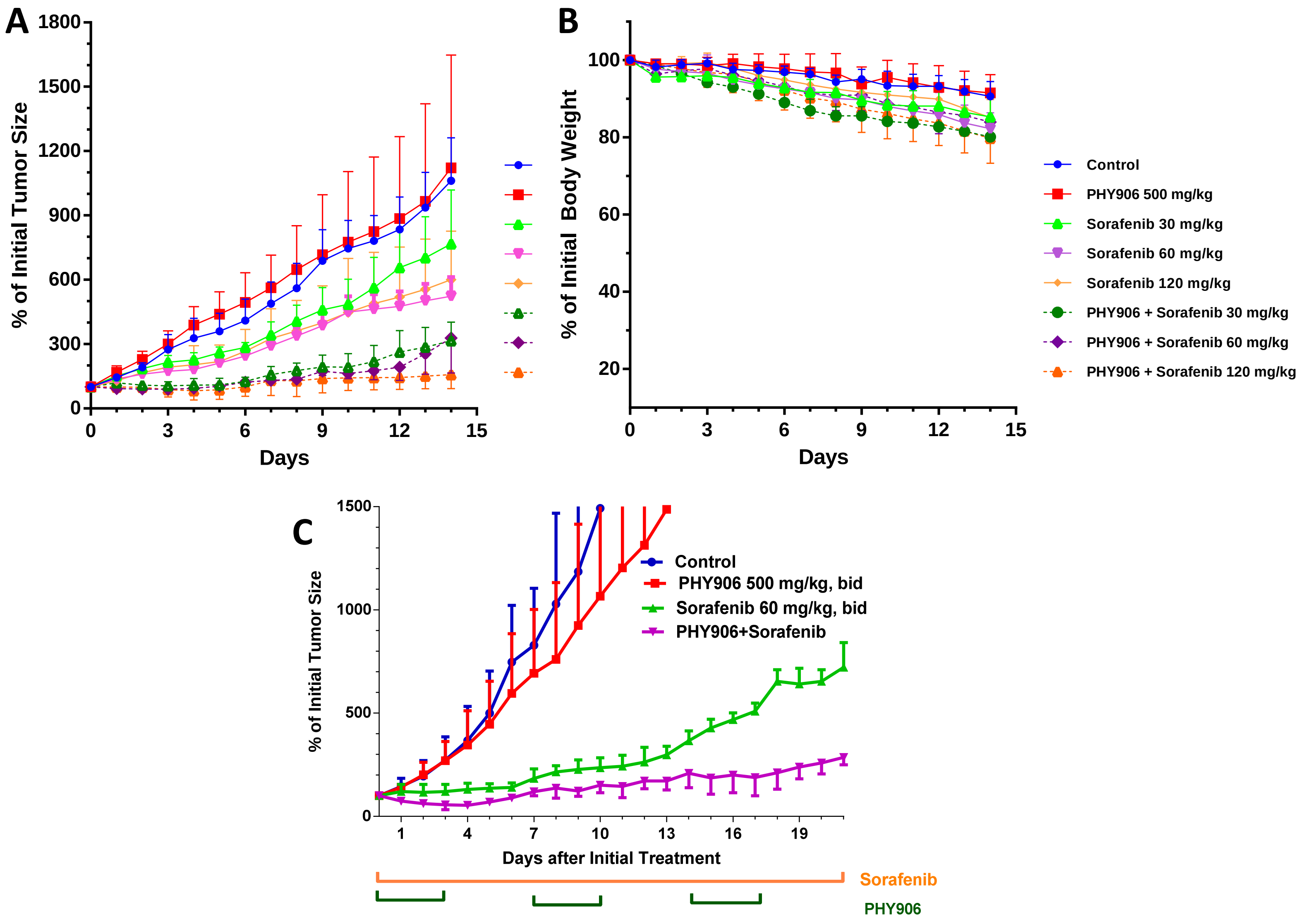

Supplementary Figure 2. Effect of PHY906 and different dosage of sorafenib on the HepG2 tumor growth (**A**)and body weight in NCR nude mice (**B**) . Sorafenib (30mg/kg, 60mg/kg or 120mg/kg as indicated in the graphs, b.i.d) was administered orally daily and PHY906 (500mg/kg b.i.d.) was administered orally from day 0 to day 3 and from day 7 to day 10 for two treatment cycles. (**C**) Effect of PHY906 and Sorafenib on the HepG2 tumor growth by three-cycle treatment. Error bars indicate standard deviations and N=5. Details of experimental procedures are given in Materials and Methods

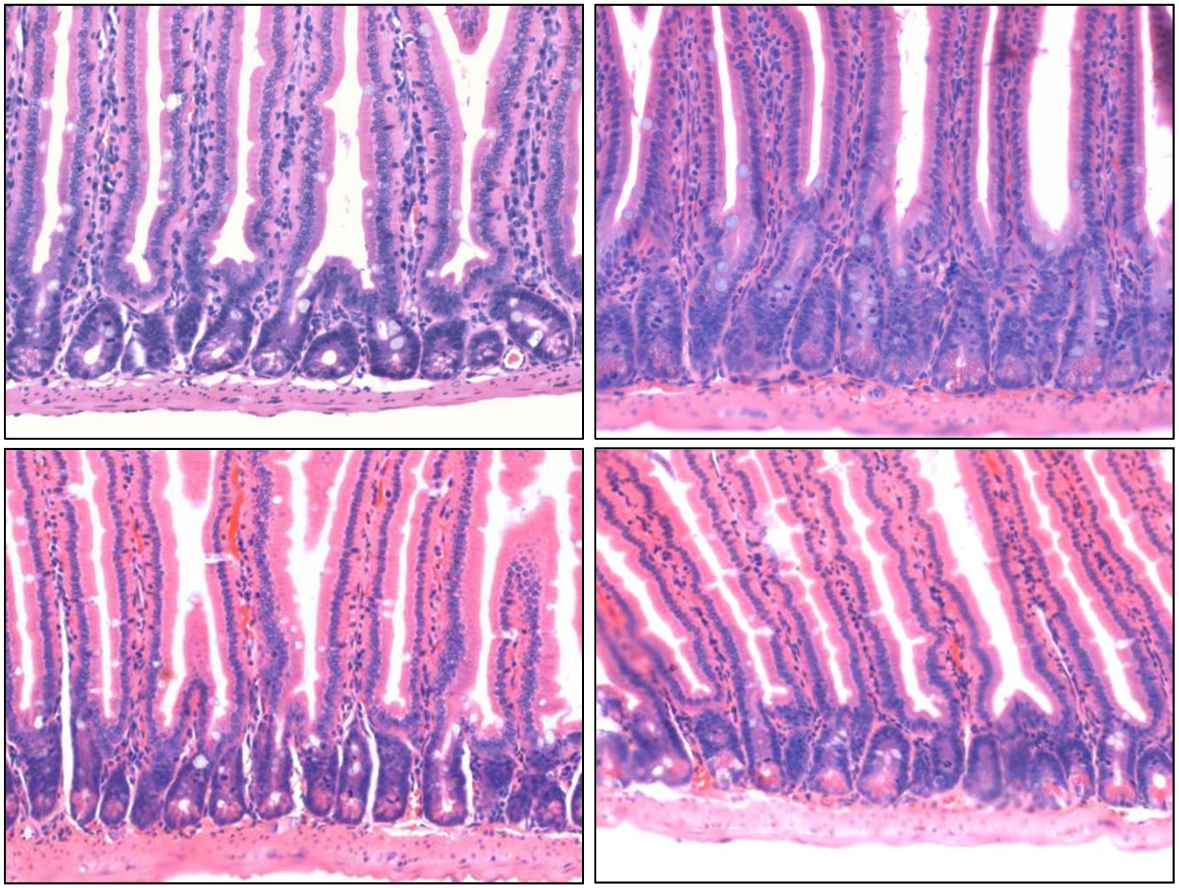

Supplementary Figure 3. Hematoxylin and eosin staining of middle jejunum sections from sample collected at day 9 after drug treatments as indicated on the above. Sorafenib (30mg/kg, b.i.d) was administered orally daily and PHY906 (500mg/kg, b.i.d.) was administered orally from day 0 to day 3 and from day 7 to day 8. Details of experimental procedures are given in Materials and Methods.

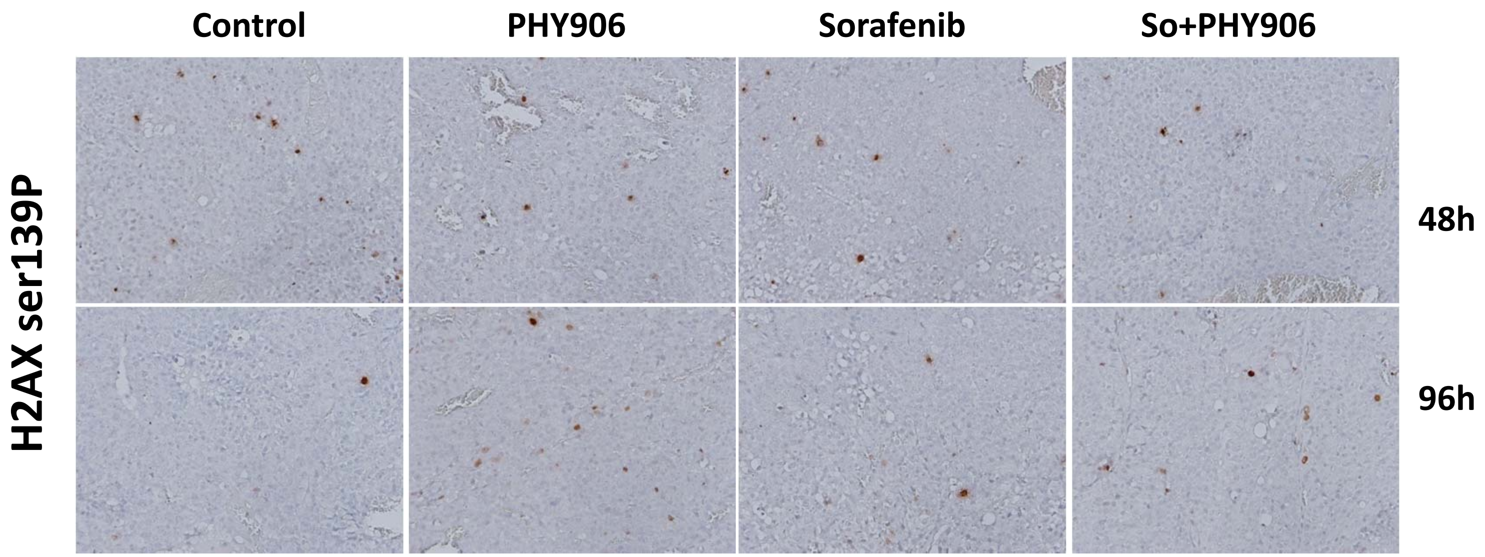

Supplementary Figure 5. Immunohistochemistry of H2AX ser139P of HepG2 tumor section tumor after the drug treatment for 48h and 96h. Sorafenib (30mg/kg, b.i.d) was administered orally daily and/or PHY906 (500mg/kg, b.i.d.) was administered orally. Details of experimental procedures are given in Materials and Methods.

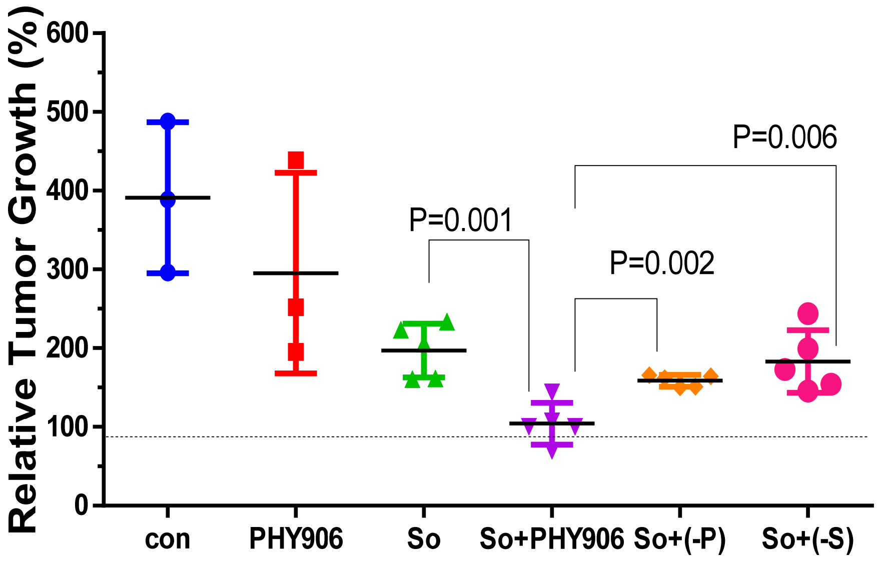

Supplementary Figure 5. Effect of PHY906, Sorafenib (So), (So+PHY906), (So+(-P)) and (So+(-S)) on HepG2 tumor growth at 96h. Number of animals is 5. Sorafenib (30mg/kg, b.i.d.) and/or PHY906, (-P), (-S) (500mg/kg, b.i.d.) were administered orally twice a day. Details of experimental procedures are given in Materials and Methods.

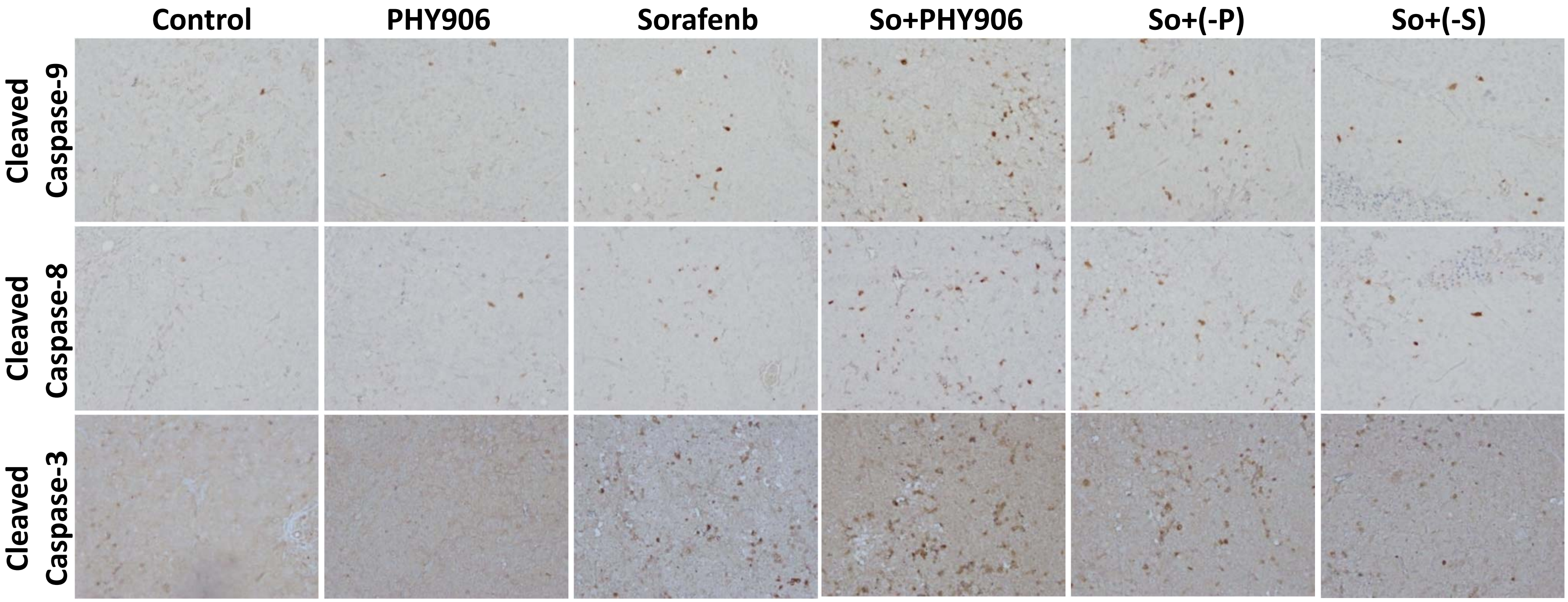

Supplementary Figure 6. Effect of PHY906, Sorafenib (So), Sorafenib+PHY906 (So+PHY906), Sorafenib+PHY906 deleted P (So+(-P)) and Sorafenib+PHY906 deleted S (So+(-S)) on the induction of apoptosis of HepG2 tumor in NCR nude mouse. Immunohistochemistry staining for cleaved caspase-3, cleaved caspase-8, cleaved caspase-9 of HepG2 tumor section after the treatments for 96h. Details of experimental procedures are given in Materials and Methods.

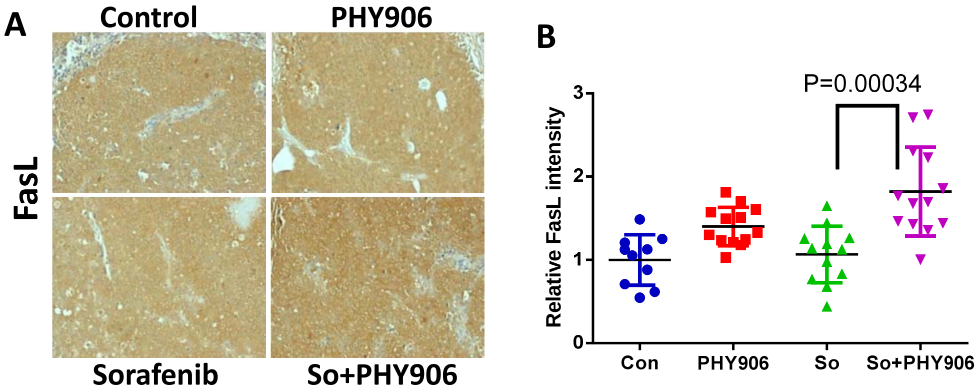

Supplementary Figure 7. Effect of PHY906 and/or Sorafenib (So) on the expression of death receptors and their ligands level HepG2 tumor in NCR nude mouse. (**A**) Immunohistochemistry staining for FasL protein of HepG2 tumor section after the drug treatment for 96h. (**B**) Quantification of immunohistochemistry staining of FasL using imaging software. (Each spot represent a mean of the intensity of FasL staining from 5 views of a tumor section and number of animals for 96h treatment group is 14). Sorafenib (30mg/kg, b.i.d) was administered orally daily and/or PHY906 (500mg/kg, b.i.d.) was administered orally. Details of experimental procedures are given in Materials and Methods.

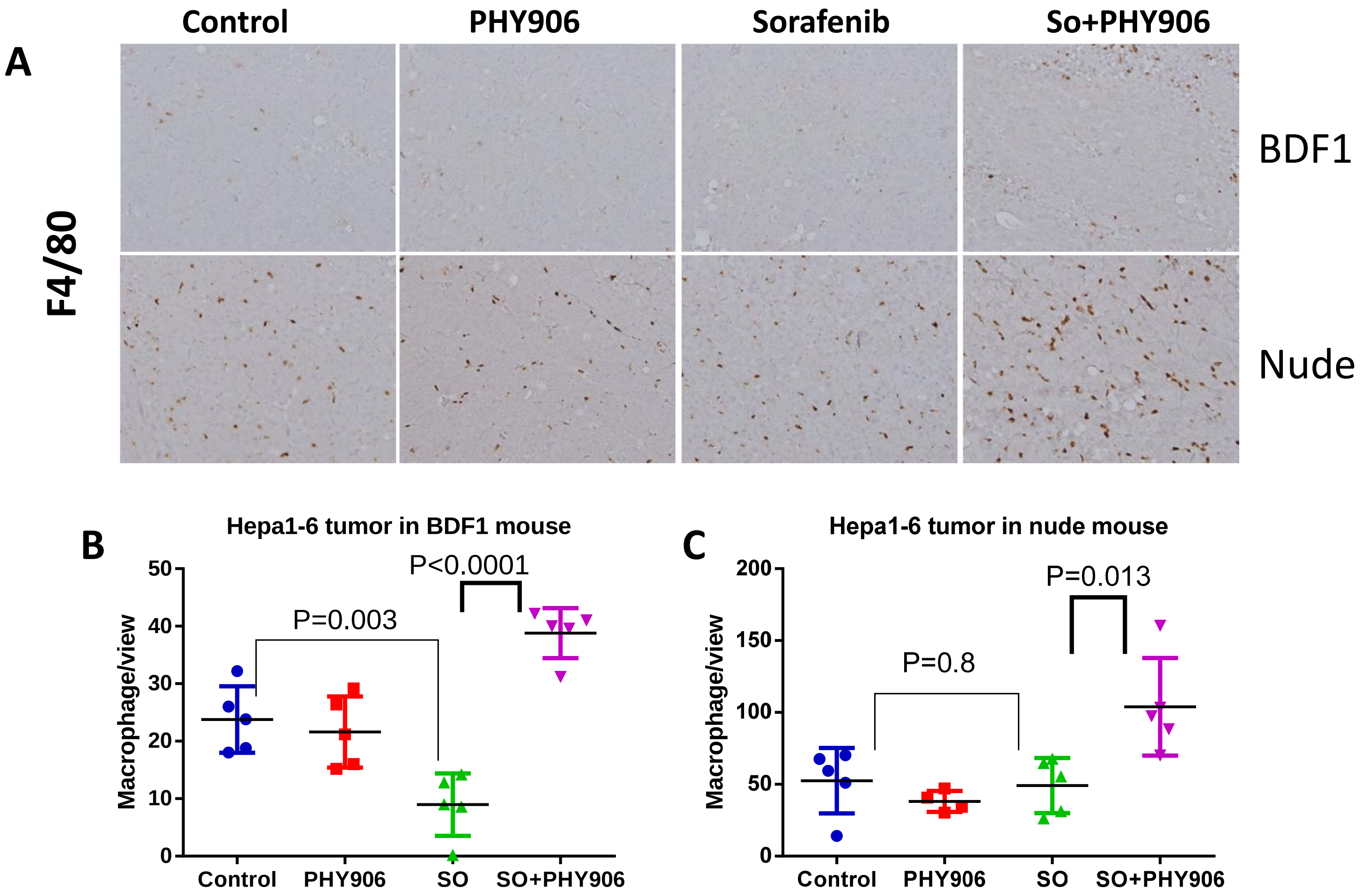

Supplementary Figure 8. Effect of PHY906 and/or Sorafenib (So) on the infiltration of macrophage of Hepa 1-6 tumor in BDF1 and NCR nude mouse. (**A**) Immunohistochemistry staining of F4/80 for macrophages of Hepa 1-6 tumor section in BDF1 mice and nude mice after 9-day treatment. The number of macrophage per each view of Hepa 1-6 tumor section in (**B**) BDF1 mice and (**c**) nude mice after 9-day treatment. Each spot represent a mean of the number of F4/80 stained cells from 4 to 5 views of each tumor section. Sorafenib (15mg/kg, b.i.d) was administered orally daily and/or PHY906 (500mg/kg, b.i.d.) was administered orally. Details of experimental procedures are given in Materials and Methods.

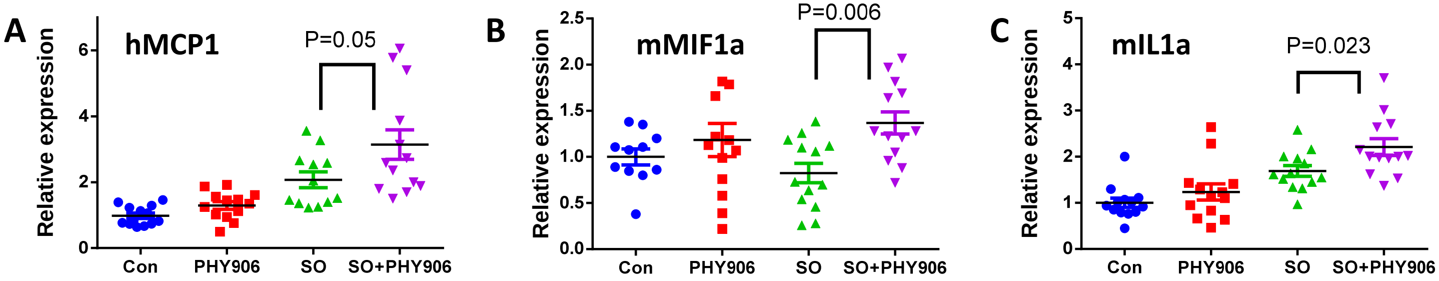

Supplementary Figure 9. The mRNA expression of human hMCP1(**A**) , mMIF1a (**B**) and mIL1a (**C**) of HepG2 tumor after the treatment of PHY906 and/or Sorafenib (So) for 96h. Each spot represent a mean of two to three different quantitative real-time PCR experiments. (triplicate samples of each; Number of animals for 96h treatment group is 14). Sorafenib (30mg/kg, b.i.d) and/or PHY906 (500mg/kg, b.i.d.) was administered orally. Details of experimental procedures are given in Materials and Methods.

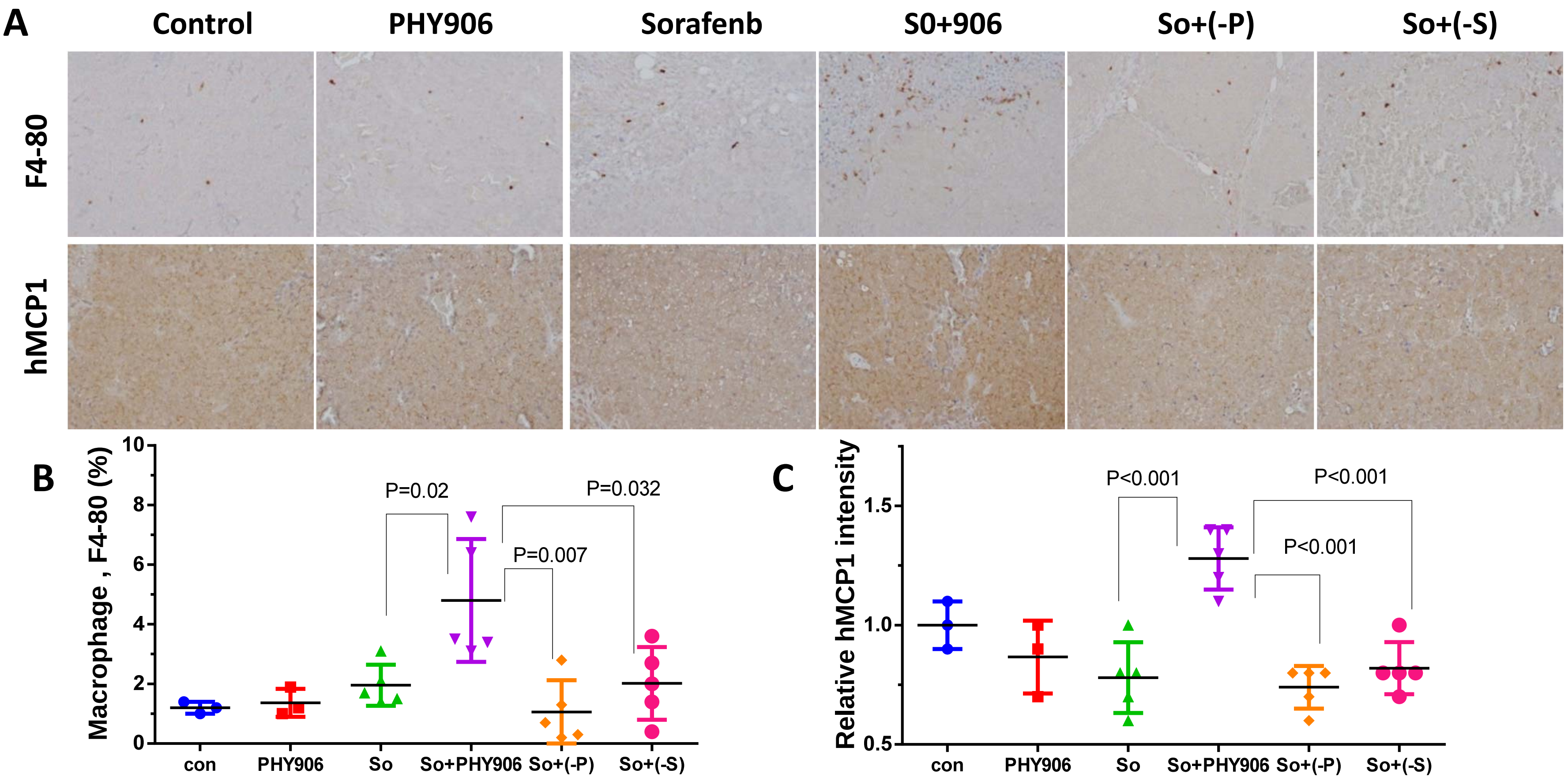

Supplementary Figure 10. Effect of PHY906, Sorafenib (So), Sorafenib+PHY906 (So+PHY906), Sorafenib+PHY906 deleted P (So+(-P)) and Sorafenib+PHY906 deleted S (So+(-S)) on the infiltration of macrophage (F4-80) and hMCP1 of HepG2 tumor in NCR nude mouse at 96h. (**A**) Immunohistochemistry of hMCP1 of HepG2 tumor section tumor after PHY906 and/or Sorafenib (So) treatment for 96h. (**B**) Percentage of F4/80 stained cell per view of HepG2 tumor section after the treatment of PHY906, Sorafenib (So), Sorafenib+PHY906 (So+PHY906), Sorafenib+PHY906 deleted P (So+(-P)) and Sorafenib+PHY906 deleted S (So+(-S)) for 96h. (**C**) Quantification of immunohistochemistry staining of hMCP1 of HepG2 tumor section after treatment with PHY906, Sorafenib (So), Sorafenib+PHY906 (So+PHY906), Sorafenib+PHY906 deleted P (So+(-P)), and Sorafenib+PHY906 deleted S (So+(-S)) for 96h. Sorafenib (30mg/kg, b.i.d.) and/or PHY906, (-P), (-S) (500mg/kg, b.i.d.) was administered orally twice daily. Details of experimental procedures are given in Materials and Methods.

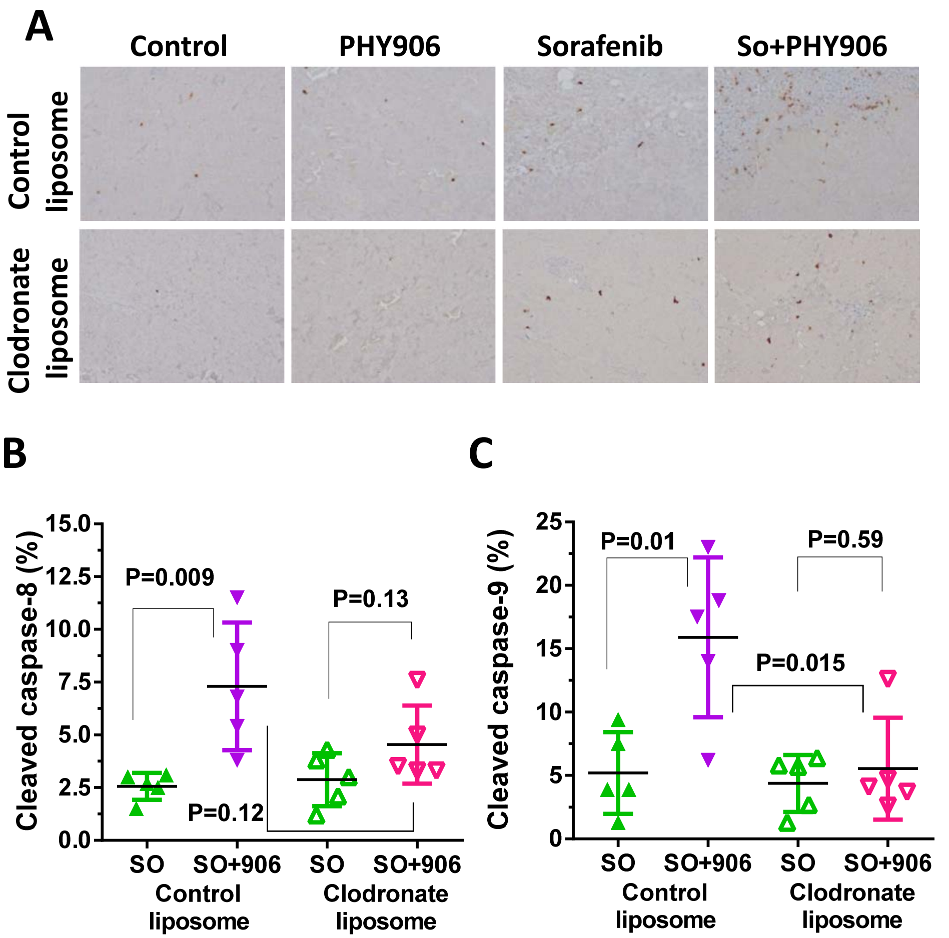

Supplementary Figure 11. Effect of clodronate liposome treatment on macrophage infiltration and apoptosis of different treatment conditions. (**A**) Immunohistochemistry of hMCP1 of HepG2 tumor section tumor after PHY906 and/or Sorafenib (So) treatment with control liposome or clodronate liposome for 96h. (**B, C**) Quantitation of immunohistochemistry staining for cleaved caspase-8, cleaved caspase-9 of HepG2 tumor sections following treatment of Sorafenib (So) or So+PHY906 for 96h. Liposomes were given using i.p. injection at day -2(0.4ml/mouse), day 0 and day 2 (0.2 ml/mouse) Each spot represents a mean of the number cleaved caspase-8 or cleaved caspase-9 stained cells from 4 to 5 views of each tumor section against total live cells (PCNA-stained) in each treatment group. Details of experimental procedures are given in Materials and Methods.

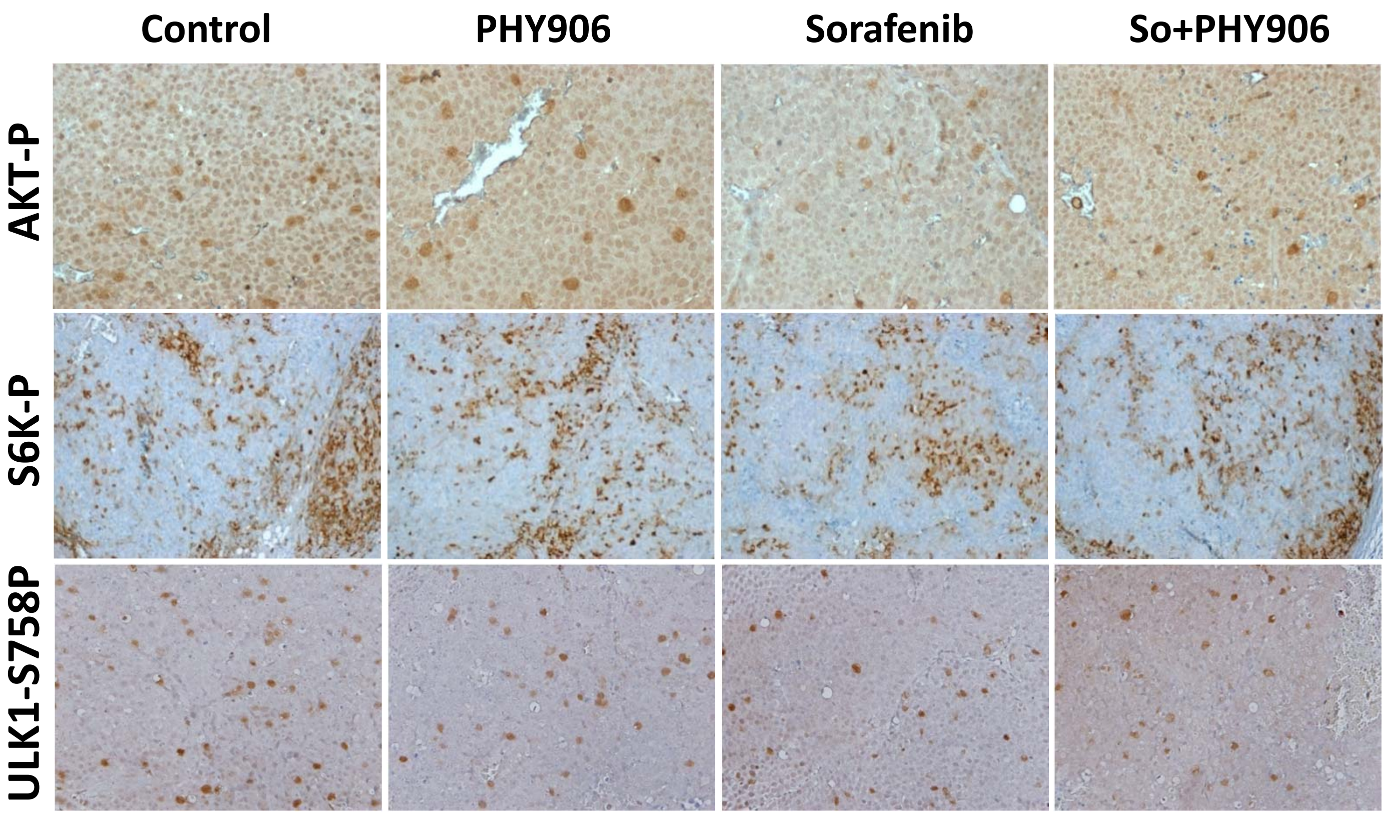

Supplementary Figure 12. Effect of PHY906 and/or Sorafenib (So) on the induction of autophagy of HepG2 tumor in NCR nude mouse. Immunohistochemistry staining for AKT-P, S6K-P and ULK1-S758P of HepG2 tumor section after the treatment of Sorafenib and Sorafenib plus PHY906 for 96h. Sorafenib (30mg/kg, b.i.d) and/or PHY906 (500mg/kg, b.i.d.) was administered orally. Details of experimental procedures are given in Materials and Methods.

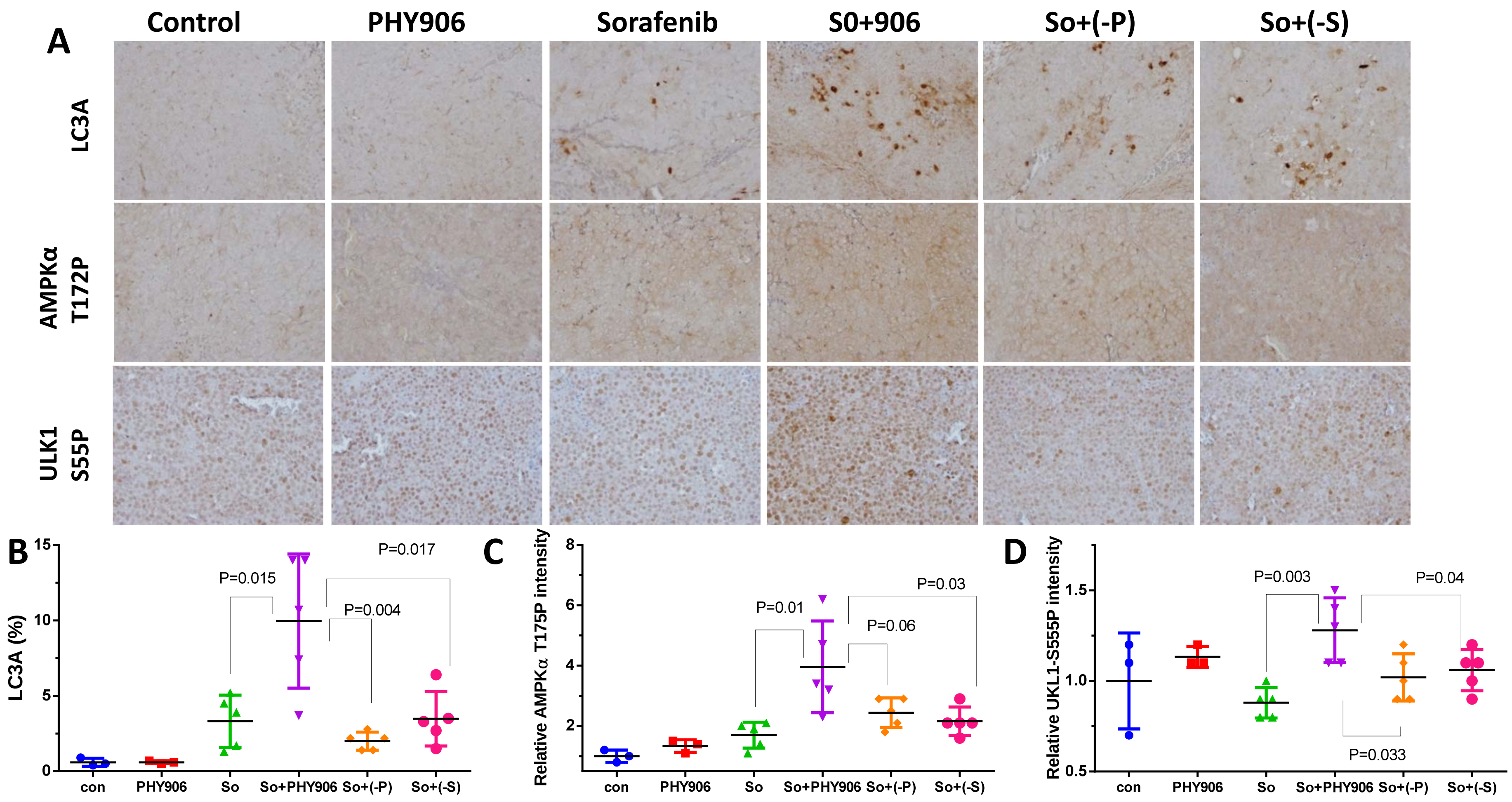

Supplementary Figure 13. Effect of PHY906, Sorafenib (So), Sorafenib+PHY906 (So+PHY906), Sorafenib+PHY906 deleted P (So+(-P)) and Sorafenib+PHY906 deleted S (So+(-S)) on LC3A, AMPKα T172P, ULK1 S555P of HepG2 tumor in NCR nude mouse at 96h. (**B**) LC3A stained cell per view of HepG2 tumor section after the treatment of PHY906, Sorafenib (So), Sorafenib+PHY906 (So+PHY906), Sorafenib+PHY906 deleted P (So+(-P)), and Sorafenib+PHY906 deleted S (So+(-S)) for 96h. Quantification of immunohistochemistry staining of (**C**) AMPKα T172P and (**D**) ULK1 S555P of HepG2 tumor section after the treatment of PHY906, Sorafenib (So), Sorafenib+PHY906 (So+PHY906), Sorafenib+PHY906 deleted P (So+(-P)), and Sorafenib+PHY906 deleted S (So+(-S)) for 96h. Sorafenib (30mg/kg b.i.d.) and/or PHY906, (-P), (-S) (500mg/kg b.i.d.) was administered orally. Details of experimental procedures are given in Materials and Methods.

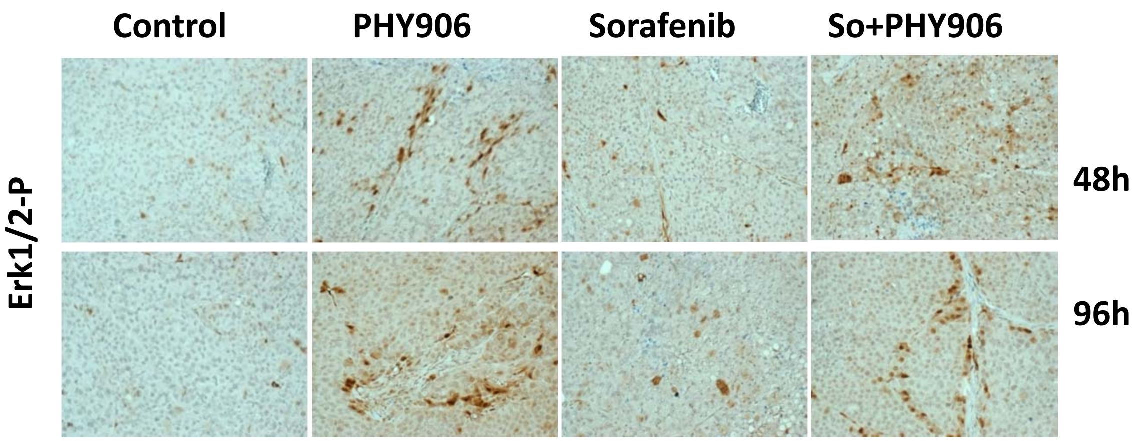

Supplementary Figure 14. Effect of PHY906 and/or Sorafenib (So) on Erk1/2 phoshporylation of HepG2 tumor in NCR nude mouse. Immunohistochemistry staining for the phosphorylated Erk1/2 (Thr202/Tyr204) of HepG2 tumor section after the drug treatment for 48h and 96h. Details of experimental procedures are given in Materials and Methods.

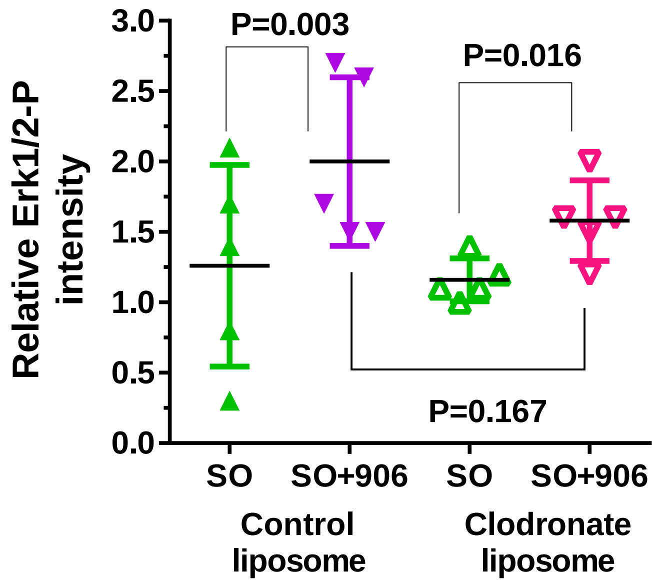

Supplementary Figure 15. Effect of clodronate liposome treatment on Erk1/2 autophagy phoshporylation of HepG2 tumor sections following treatment of Sorafenib (So) or So+PHY906 for 96h. Liposome were given using i.p. injection at day -2, day 0 and day 2. Each spot represents a mean of the number LC3A stained cells or the intensity of brown color from 4 to 5 views of each tumor section in each treatment group. Details of experimental procedures are given in Materials and Methods.

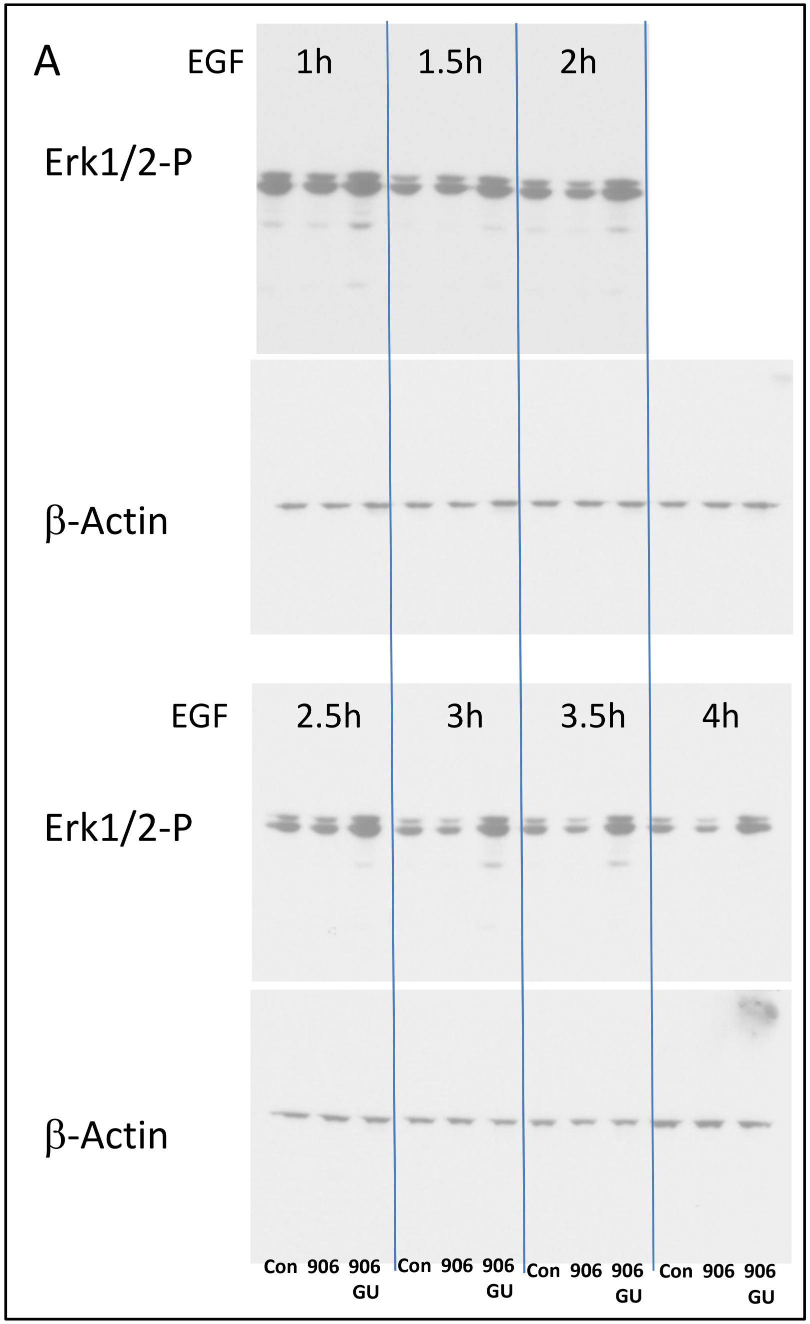

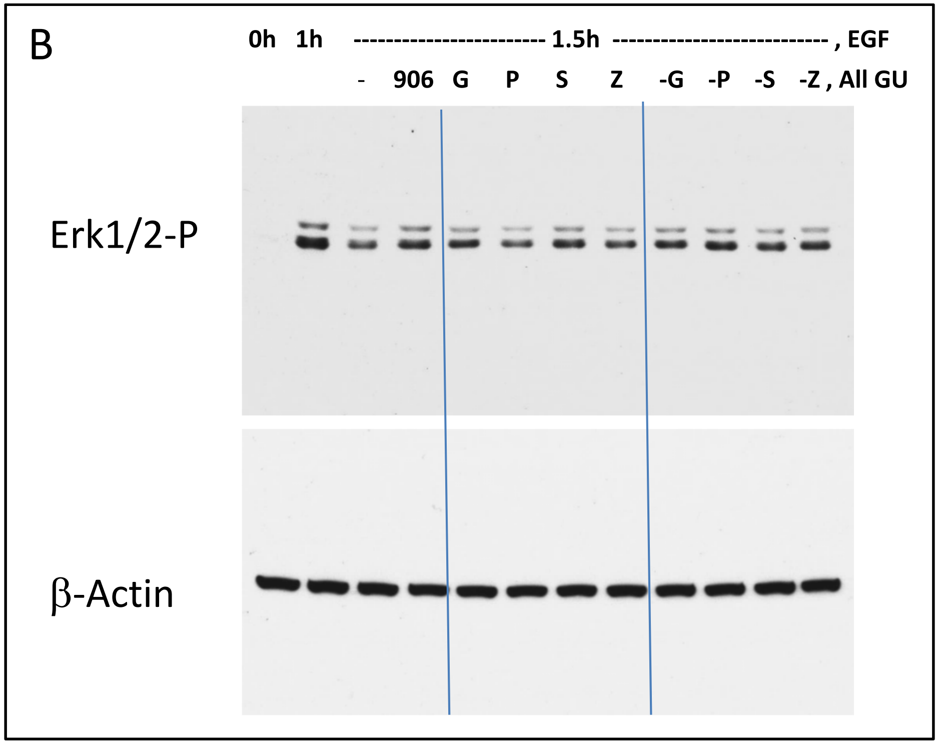

Supplementary Figure 16. Effect of PHY906 on dephosphorlayion of ERk1/2. **A**) Western blotting analysis for the effect of PHY906 or *E.coli* β-glucuronidase treated PHY906 (500µg/ml) on dephosphorylation rate of Erk1/2 in HepG2 cells following stimulation with EGF (50ng/ml). β-actin was used as the loading control for normalization. (**B**) Western blotting analysis for the effect of *E.coli* β-glucuronidase treated PHY906 (500µg/ml), equivalent concentration of single herbs (G, P, S, Z), or equivalent concentration of a one herb deleted formula (-G, -P, -S, -Z) on dephosphorylation rate of Erk1/2 in HepG2 cells following stimulation with EGF (50ng/ml) .

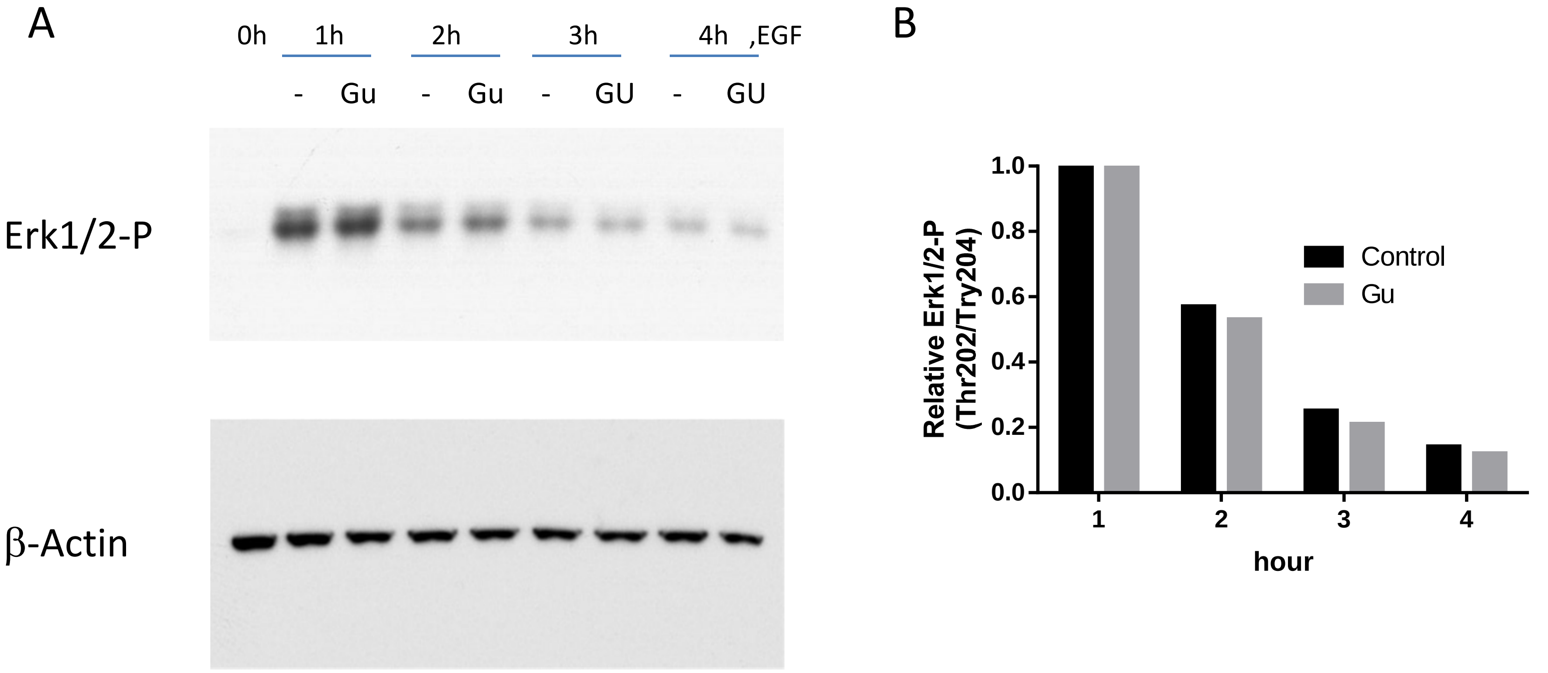

Supplementary Figure 17. Effect of *E.coli* β-glucuronidase *(*Gu*)* on dephosphorlayion of ERk1/2. **A**) Western blotting analysis for the effect of PHY906 or *E.coli* β-glucuronidase treated PHY906 (500μg/ml) on dephosphorylation rate of Erk1/2 in HepG2 cells following stimulation with EGF (50ng/ml). β-actin was used as the loading control. (**B**) Quantification of the Western blot results for the phosphorylated Erk1/2 (Thr202/Tyr204). Details of experimental procedures are given in Materials and Methods.

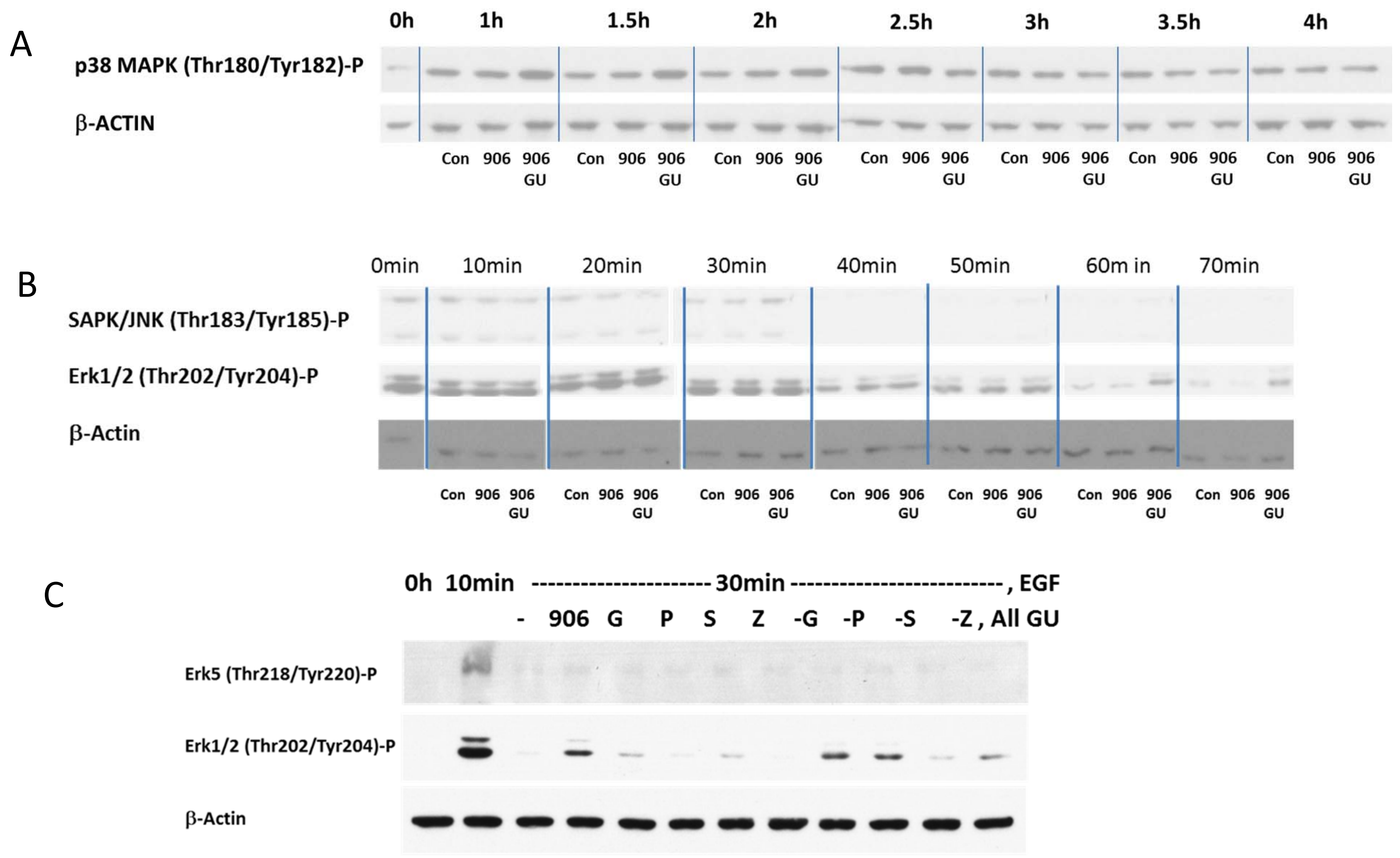

Supplementary Figure 18. Effect of PHY906 on p38 MARK, SAPK/JNK, ERK5, ERK1/2 phoshporylation of HepG2 cell in culture. (**A**) Western blotting analysis for the effect of PHY906 or *E.coli β-glucuronidase* treated PHY906 (500μg/ml) on dephosphorylation rate of p38 MARK (Thr180/Tyr182) in HepG2 cells after stimulated using EGF (50ng/ml) for 1h. β-actin was used as the loading control. (**B**) Western blotting analysis for the effect of PHY906 or *E.coli β-glucuronidase* treated PHY906 (500μg/ml) on dephosphorylation rate of SAPK/JNK(Thr183/Tyr185) in HepG2 cells after stimulated using H2O2(1mM) for 1h. β-actin was used as the loading control.(**C**) Western blotting analysis for the effect of *E.coli β-glucuronidase* treated PHY906 (500μg/ml), equivalent concentration of single herbs (G, P, S, Z), or), equivalent concentration of one herbs deleted formula (-G, -P, -S, -Z) on dephosphorylation rate of ERk5(Thr218/Tyr220) and Erk1/2(Thr202/Tyr204) in HepG2 cells after stimulated using EGF (50ng/ml) for 10min. Details of experimental procedures are given in Materials and Methods.
